# Histological Evaluation of Diabetic Neurodegeneration in the Retina of Zucker Diabetic Fatty (ZDF) Rats

**DOI:** 10.1038/s41598-017-09068-6

**Published:** 2017-08-21

**Authors:** Klaudia Szabó, Anna Énzsöly, Bulcsú Dékány, Arnold Szabó, Rozina I. Hajdú, Tamás Radovits, Csaba Mátyás, Attila Oláh, Lenke K. Laurik, Gábor M. Somfai, Béla Merkely, Ágoston Szél, Ákos Lukáts

**Affiliations:** 1 Department of Anatomy, Histology and Embryology, Semmelweis University, Budapest, H-1085 Hungary; 2 Department of Ophthalmology, Semmelweis University, Budapest, H-1085 Hungary; 3 Heart and Vascular Center, Semmelweis University, Budapest, H-1085 Hungary

## Abstract

In diabetes, retinal dysfunctions exist prior to clinically detectable vasculopathy, however the pathology behind these functional deficits is still not fully established. Previously, our group published a detailed study on the retinal histopathology of type 1 diabetic (T1D) rat model, where specific alterations were detected. Although the majority of human diabetic patients have type 2 diabetes (T2D), similar studies on T2D models are practically absent. To fill this gap, we examined Zucker Diabetic Fatty (ZDF) rats - a model for T2D - by immunohistochemistry at the age of 32 weeks. Glial reactivity was observed in all diabetic specimens, accompanied by an increase in the number of microglia cells. Prominent outer segment degeneration was detectable with changes in cone opsin expression pattern, without a decrease in the number of labelled elements. The immunoreactivity of AII amacrine cells was markedly decreased and changes were detectable in the number and staining of some other amacrine cell subtypes, while most other cells examined did not show any major alterations. Overall, the retinal histology of ZDF rats shows a surprising similarity to T1D rats indicating that despite the different evolution of the disease, the neuroretinal cells affected are the same in both subtypes of diabetes.

## Introduction

Diabetes and its related complications including retinopathy cause a great social and economic burden globally, with a pandemic-like increase in the number of patients affected. Today, 415 million people are estimated to have diabetes worldwide^1^, among them type 2 diabetes (T2D) accounts for approximately 90% of the cases. Diabetic retinopathy is affecting one-third of the people living with diabetes^1^ with approximately 5 million cases of blindness worldwide that can be attributed to this disease^2^.

The pathophysiology of diabetic retinopathy is complex. Besides the clinically detectable vascular alterations like microaneurysms, haemorrhages, vascular leakage and neovascularization it also includes neural retinal components^3^. Neuroretinal pathology may be represented by functional deficits like abnormalities of electroretinographic (ERG) data and contrast sensitivity. Such impairments have been demonstrated in both animal models and human diabetic patients earlier than the appearance of funduscopically detectable vascular signs^4, 5^. Furthermore, these functional alterations may progress together with vascular retinopathy, or may even predict its progression^6^. Exploring the underlying mechanisms may open new perspectives in understanding the pathomechanism of the disease.

Similarly to other reports^7–12^ our previous studies^13, 14^ have demonstrated that in experimentally induced type 1 diabetic (T1D) rats, several cell types of the retina, including astrocytes, Müller glia cells, photoreceptors, retinal pigment epithelium (RPE), amacrine and ganglion cells are already affected, even prior to significant apoptosis or clinically detectable vasculopathy.

In contrast to these observations, there is much less information on the early neuroretinal alterations in T2D models^15^. As the vast (and increasing) majority of human diabetic patients have T2D, it could be of particular interest.

Several T2D rat models such as ZDF, OLETF, SDT rats etc^16^. has become available in the past decades. Most of these models carry mutations in a single or multiple genes, related to obesity or insulin resistance, and recapitulate some, but never all features of human T2D^16–18^. Due to a mutation in the leptin receptor gene, ZDF *fa/fa* rats become obese, develop hyperglycaemia, hyperlipidaemia and early hyperinsulinemia on a high-fat diet by 12 weeks of age. By postnatal week 20, with continuously high glucose levels, ZDF rats become insulinopenic with markedly reduced insulin levels^19^. Thus, diabetes in ZDF rats has common general metabolic characteristics with the human T2D^20^.

Although the development of vasculopathy in the retina is not specific to either T1D or T2D and there are no noteworthy clinical differences regarding the vascular lesions themselves^21, 22^, early retinopathy could be a different issue. Elevated insulin levels, insulin resistance, obesity and the initially moderate rise of blood glucose levels in T2D are amongst the factors that need to be considered during evaluation. Insulin, for example, is considered as one of the key factors in the development and surviving of the cone and rod outer segment^23, 24^. It is possible therefore, that T2D rat models will show differences in the type of retinal cells affected or in the rate of damage. Comparing the retinas of T1D and T2D rat models could help to elucidate the role of insulin signalling in the development of histopathological changes in the retina.

In order to examine this possibility, in this report, we aimed to give a detailed qualitative and quantitative description on the early histopathological changes in the retina of ZDF rats and compare our results with previous studies dealing with experimentally induced T1D rats^13, 14^. Overall, we demonstrate here that the retinal histopathology of ZDF rats shows a surprising similarity to streptozotocin (STZ)-induced T1D rats. Thus, despite the different evolution of the disease, the neuroretinal cells affected are the same in both subtypes of diabetes, suggesting that most of the changes are probably the consequences of the high glucose levels whereas altered insulin signalling may play a less prominent role in the development of neural alterations. Preliminary results of this study have already been published in the form of an abstract^25^.

## Results

### Body weight and blood glucose levels

**Figure 1:**
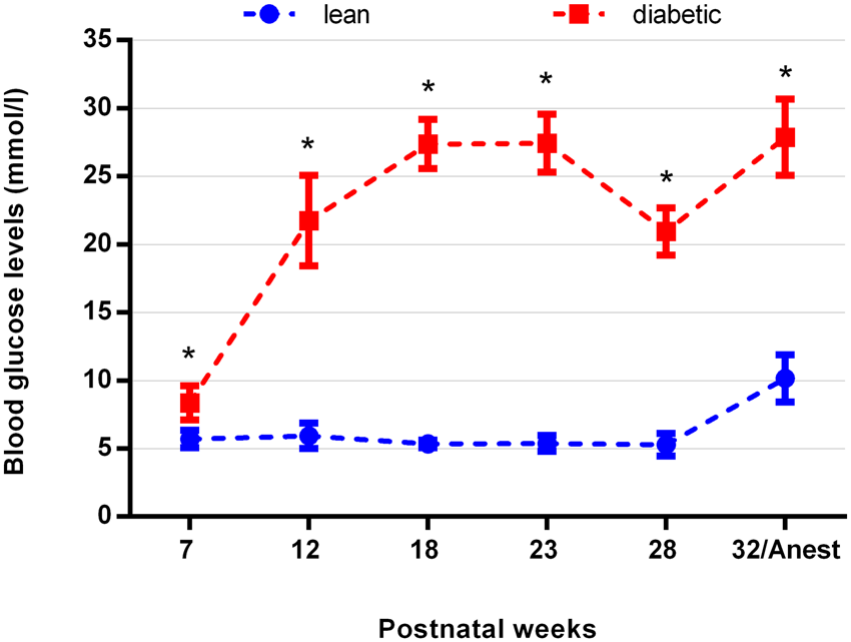
Blood glucose levels of lean and diabetic animals measured in different postnatal ages. From the 7^th^ week till the end of the observation period (time of anaesthesia, 32^nd^ week (32/Anest)) blood glucose levels were significantly higher in diabetic specimens compared to controls. ^*^p < 0.05.

There was no significant difference in body weight between lean (421.3 ± 21.6 g) and diabetic animals (400.3 ± 50.2 g) at the time of euthanasia. The blood glucose levels remained normal throughout the complete observation period for lean animals (except for the values during anaesthesia) while for diabetic specimens they were significantly higher even at week 7, continued to rise till week 12 and remained elevated thereafter till euthanasia. Changes of blood glucose levels are demonstrated in Fig. 1. The blood glucose levels detected by us were in the middle of the “expected” range published by the supplier (Charles Rivers^26^). Detailed list of the appropriate p values and the results of the comparison within the same group at different postnatal ages are given in Supplementary material, Table S1.

### Apoptosis and thickness measurements

**Figure 2:**
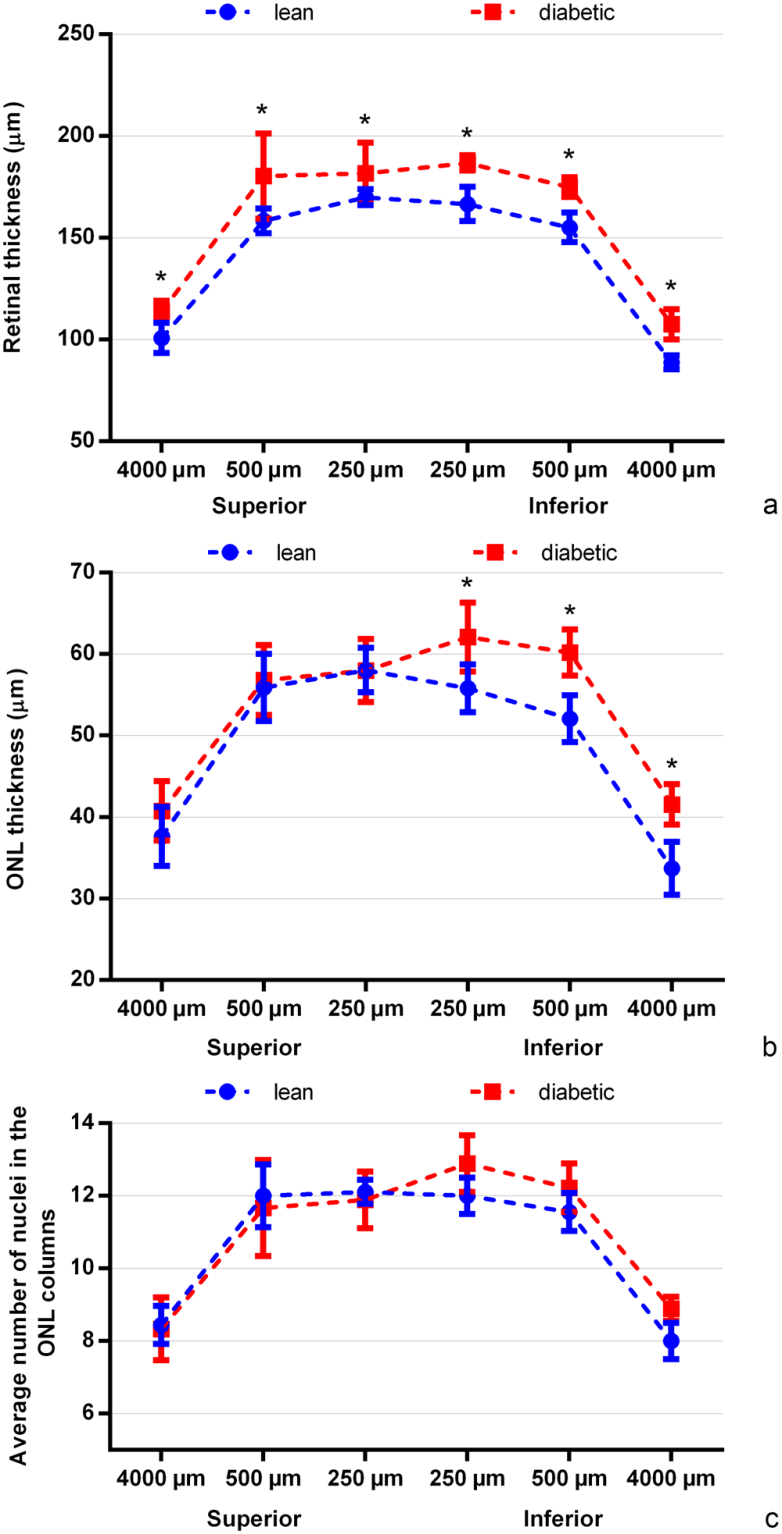
Comparison of the thickness of the full retina (ILM-OLM distance), the ONL layer alone, and the average number of nuclei in an ONL column between diabetic and lean animals. Samples were taken 250 µm, 500 µm and 4000 µm superiorly and inferiorly from the optic nerve head. *indicate a significant increase in the parameters measured in diabetic specimens. There was no significant difference in the number of nuclei in the ONL columns in any region examined. ILM: inner limiting membrane, OLM: outer limiting membrane, ONL: outer nuclear layer, ON: optic nerve, ^*^p < 0.05.

There was a slight but significant increase in thickness of the whole retina (ILM-OLM) in all retinal positions and in ONL thickness in the inferior retinal half in diabetic ZDF rats compared to controls (Fig. 2a,b), probably due to oedema formation. Detailed list of the appropriate p values and the results of the comparison within the same group at different retinal locations are given in Supplementary material, Tables S2 and S3. Retinal thickness may remain normal or increase even in case of significant cell loss due to intracellular oedema formation that is known to occur in diabetes^27^. In order to exclude this possibility, we also attempted two other methods to evaluate the actual number of cell types. (1) In the ONL, nuclei are typically arranged in columns on well oriented sections; therefore, we counted the average number of nuclei per column in the same regions where the thickness measurements were performed. No significant difference was detectable (Fig. 2c, Table S4). (2) We also counted the number of immunohistochemically labelled elements on vertical sections. The results are detailed below in the subsequent paragraphs but in brief, they also exclude any major cell loss.

In line with this, we detected no increase in the number of apoptotic cells in TUNEL assays. Representative pictures of TUNEL labelled control and diabetic retinas are shown in Supplementary material, Fig. S5. The average number of apoptotic cells in the control specimens was 4.1 ± 3.05 per retina vs. 2.4 ± 1.6 in diabetic specimens, significantly lower in diabetic specimens (p: 0.02). Overall, we can conclude that the diabetic retinas were inspected prior to significant apoptosis.

### Glial response

**Figure 3:**
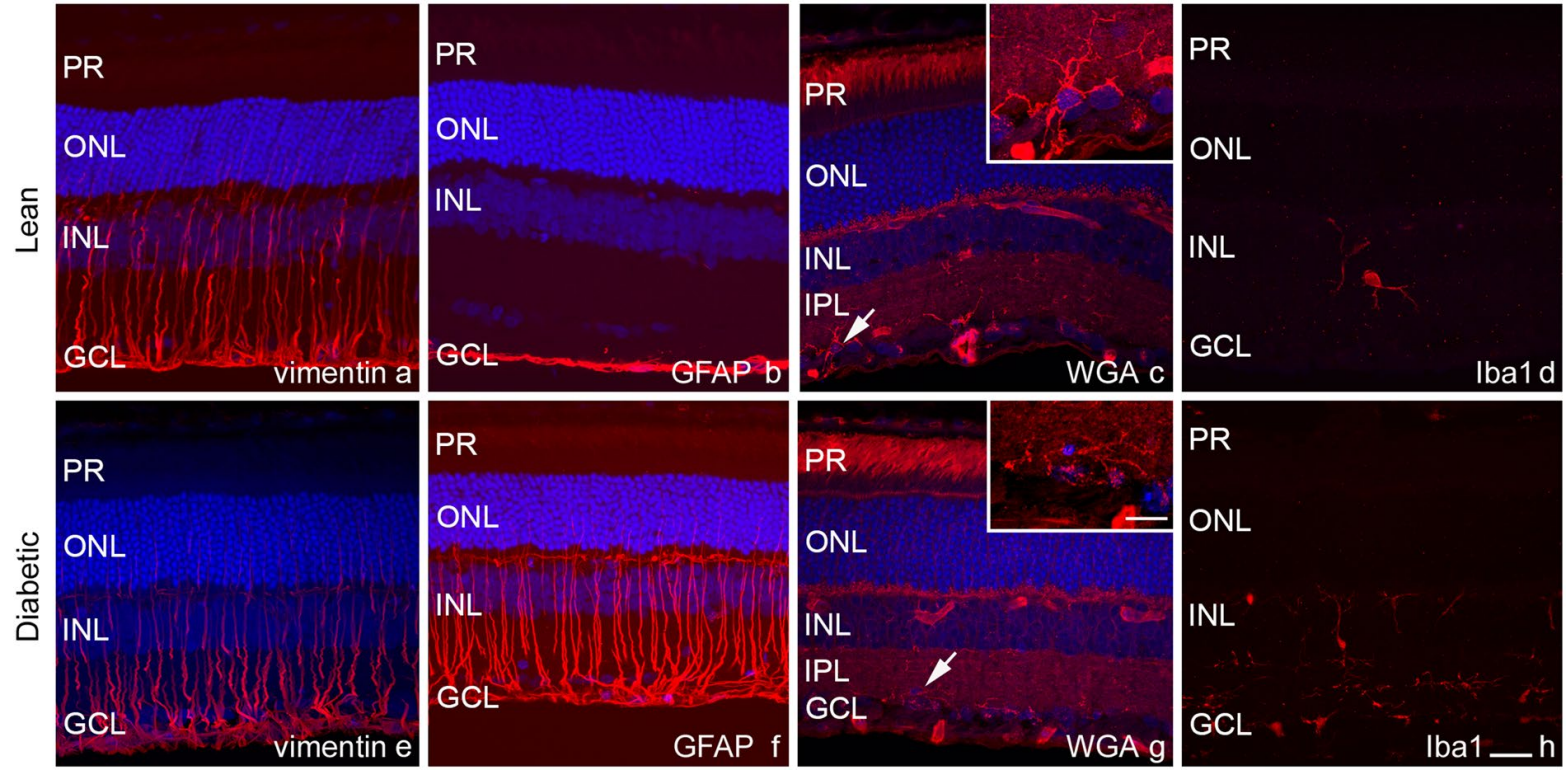
Glial responses in diabetes. Upper row shows control, lower row diabetic specimen. Müller glia cells were analysed using vimentin and GFAP antibodies. A marked increase in GFAP expression (**b**,**f**) but no major change in vimentin (**a**,**e**) labelling was evident. Microglia activation was analysed with WGA lectin and Iba1 antibody. Besides microglia cells WGA (**c**,**g**) labels the interphotoreceptor matrix of rods, blood vessels, and the plexiform layers. The microglia cells stained showed similar morphology (arrows on c and g, the same cells on high magnification on the insets). With Iba1 antibody (**d**,**h**) an approximately 2.5-fold increase in the number of microglia cells was detectable in diabetic specimen without evident signs of microglia activation. DAPI is used as a nuclear staining (**a**–**c** and **e**–**g** in blue). PR: photoreceptor layer, ONL: outer nuclear layer, INL: inner nuclear layer, IPL: inner plexiform layer, GCL: ganglion cell layer. *Bar*: 20 μm and 10 μm for the insets.

Müller glia cells were analysed using antibodies against cell type specific cytoskeletal elements: GFAP and vimentin. Under physiological conditions GFAP intermediate filaments are detectable only near the ILM, in the end feet of the Müller glia cells and in astrocytes of the nerve fibre layer^7^. Vimentin on the other hand stains the whole Müller cells even under control conditions^28^. In agreement with our previous study about STZ-induced diabetic retinopathy^14^ we found a major increase in GFAP expression, with a complete staining throughout the full length of Müller cells but no evident change in vimentin labelling in the ZDF retinas compared to controls. The change in GFAP labelling was prominent all over the retina. Results are demonstrated on Fig. 3a,e for vimentin, b and f for GFAP.

To analyse the microglia population the slides were labelled with WGA lectin and Isolectin B_4_. With WGA, besides the dominant labelling of blood vessels and the interphotoreceptor matrix of rods, we found several star shaped cells exclusively in the inner retina with long branching processes typical for resting microglia (Fig. 3c,g). No noteworthy difference was detected between diabetic and lean rats. Isolectin B_4_ (besides a faint but visible staining of cones and the prominent staining of blood vessels) also stained a population of microglia-like cells with similar number and morphology without any detectable change under diabetic conditions (not shown). With these two lectins - labelling mostly long processes but not the complete cell body of microglia cells - the precise quantification was impossible. In order to ensure that all microglia cells are labelled and counted, we stained some sections with Iba1 antibody, recognizing both resting and activated microglia cells. The overall number of cells per sections were calculated and revealed an approximately 2.5-fold increase in diabetic specimens compared to controls (111 ± 8.6 in control vs. 297.8 ± 37.5 cells per section in diabetic specimens, p: 0.0002). The cells showed no evident sign of microglia activation (Fig. 3d,h): there was no change in overall morphology and labelled cells never appeared in the ONL. Recent publications however indicate that in the retina microglia activation may not necessarily coincide with altered morphology^29^.

### Photoreceptor degeneration

Different cone populations can be distinguished with immunocytochemistry. In control rat retinas, the majority of cones are middle wavelength sensitive (M-cones), while approximately ten percent belongs to the short wavelength sensitive type (S-cones)^30, 31^. A few cones in the peripheral retina contain both photopigments (dual cones)^32^.

**Figure 4:**
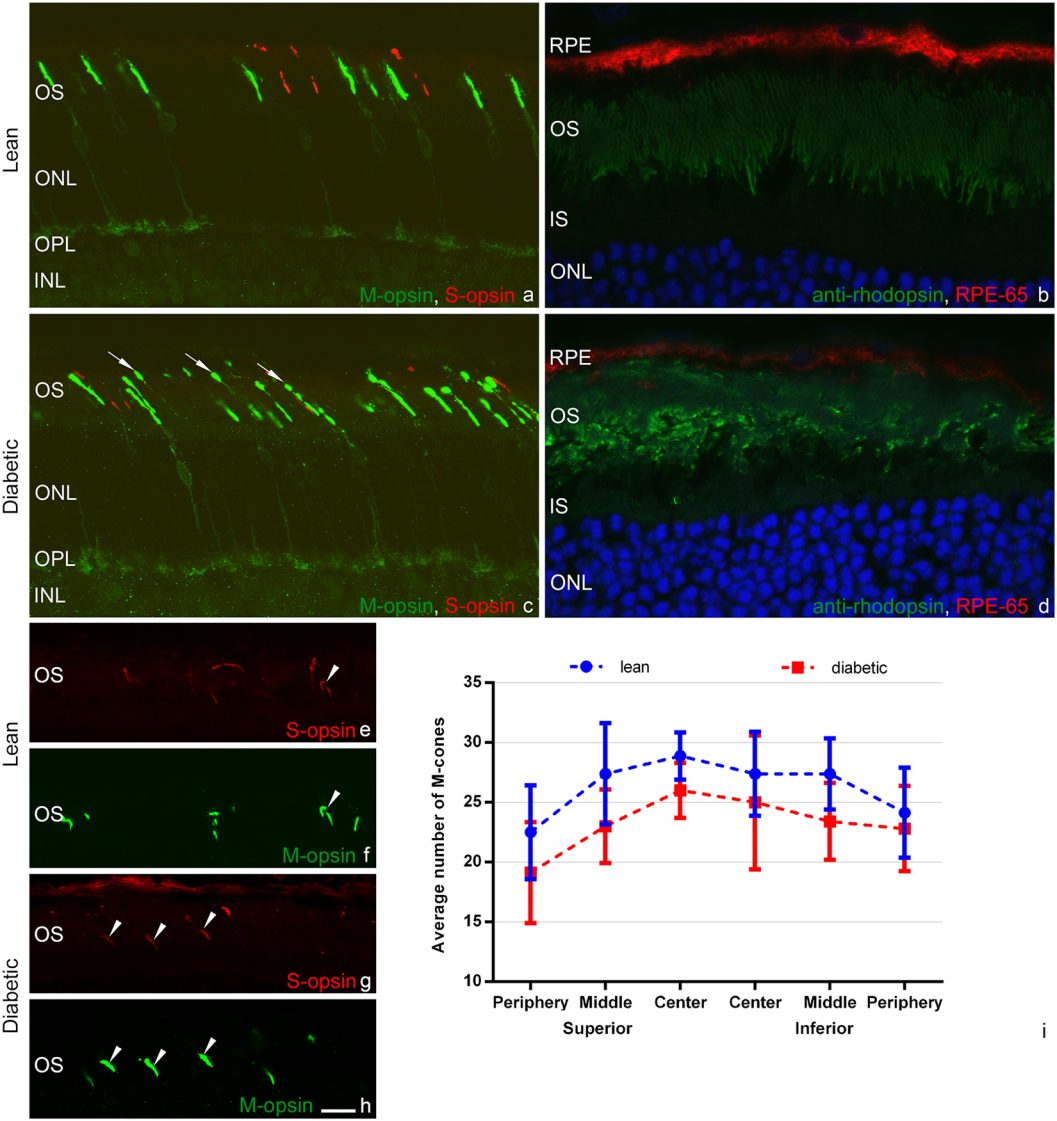
Outer segment degeneration of rods and cones with an increase in the number of dual cones in the peripheral retina in diabetes. Cone populations were labelled by AB5405 polyclonal antibody recognizing M-opsin (**a**,**c** and **e–h**, in green) and OS-2, staining S-opsin (**a**,**c**, **e–h**, in red). Note the evident outer segment degeneration in diabetes (**c**, arrows) compared to controls (**a**), without any major decrease in the number of M-cones in any region examined (**i**). In the peripheral retina of lean ZDF rats only a few (**e**,**f**), while in diabetic specimens near the ora serrata almost all M-cones co-label with S-opsin (**g**,**h**). Some dual cones are marked by arrowheads. Rod outer segments labelled with anti-rhodopsin antibody (in green) also show severe morphological degeneration in diabetic specimens (**d**) compared to controls (**b**). On the same pair of pictures a visible decrease in the intensity of RPE-65 staining of the pigmented epithelium is also evident (in red). DAPI was used for nuclear staining (in blue). RPE: retinal pigment epithelium, OS: outer segment, IS: inner segment, ONL: outer nuclear layer, OPL: outer plexiform layer, INL: inner nuclear layer. *Bar*: 10 μm for (**a**–**d**) and 15 μm for (**e–h**).

**Figure 5:**
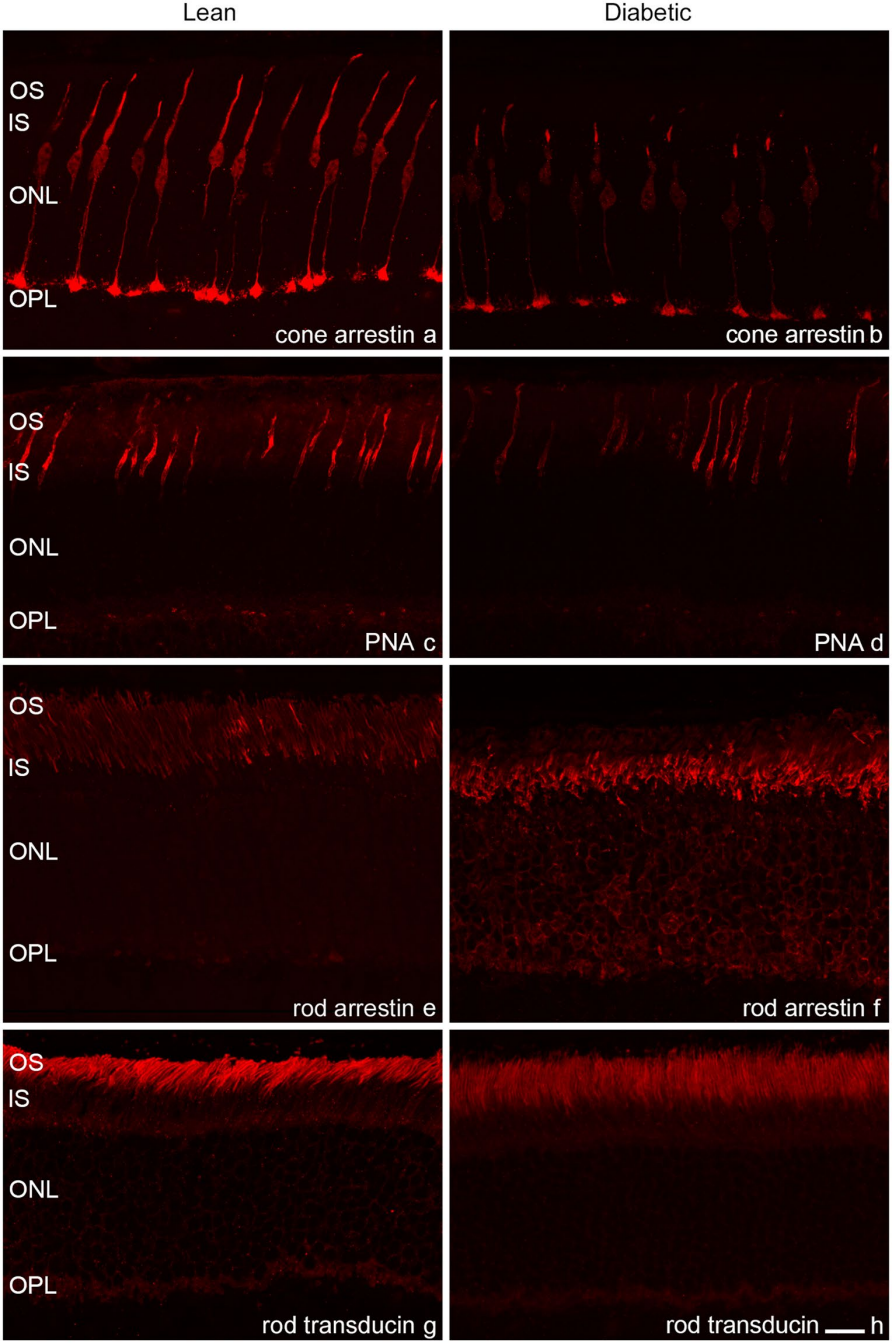
Elements of the phototransduction cascade and cone interphotoreceptor matrix in diabetes. Severe outer segment degeneration of cones was also evident with cone arrestin antibody ((**a)**: control, (**b)**: diabetic specimens), while PNA staining of the interphotoreceptor matrix of the cones did not show any major difference ((**c**): control, (**d**): diabetic). When comparing the expression of the regulatory elements of the rod phototransduction cascade, only minor differences were detected. Whereas in most retinal regions the expression pattern was similar to control (**e**), with rod arrestin a protein mislocalization to the perikarya was detectable (**f**) but only in some regions showing the most deteriorated outer segment morphology. No such change was evident for rod transducin ((**g**): control, (**h**): diabetic). OS: outer segment, IS: inner segment, ONL: outer nuclear layer, OPL: outer plexiform layer, *Bar*: 20 μm.

There was no difference in labelling intensity of M- and S-cones in diabetic specimens (Fig. 4c) compared to controls (Fig. 4a). Regarding morphology however, there was a remarkable outer segment degeneration, manifested by the fragmentation of outer segments with thin stalks connecting the fragments (Fig. 4c – some of the fragmented outer segments are marked by arrows). As an advantage of using relatively thick frozen sections, with a thickness of approximately 20 micrometres – the range that equals the length of the cone outer segments – it was relatively easy to follow most outer segments completely in all sections with confocal microscopic scans, even if the section was not fully vertical. Checking concomitant focal planes, it was possible to ensure that the fragments of the degenerated outer segments belonged to the same element. Representative confocal stacks from lean and diabetic retinas are available for download at https://drive.google.com/open?id=0ByKM9hoX0qWxRV8zZGt1ZDcxSUU. The majority of M-cones were affected all over the retina. This is in full agreement with our previous results obtained in STZ-induced diabetic rats^13^. In order to further analyse the outer segment changes in ZFD rats, we applied an antibody against cone arrestin - a functional protein in the inactivation phase of the phototransduction cascade. Although the degenerated profile of cone outer segments was evident, we found no change in the staining intensity of this antibody (Fig. 5a,b).

Due to the prominent outer segment degeneration, the precise quantification of cone numbers on sections was not feasible. We only attempted counting on a limited number of sections. The results of M-cone counts in different retinal regions were given in detail in Fig. 4i. Due to the low number of sections and the uncertainty of outer segment recognition no statistical evaluation was performed. We can rule out however any major loss of cone photoreceptor cells.

In the peripheral retina, there was a sharp increase in the number of dual cones, where almost all cones coexpressed both M- and S-opsins in diabetic animals (Fig. 4e–h). A few dual cones were also detected in central regions in diabetic specimens, albeit in much smaller numbers. Dual cones were reliably identified with all antibody combinations used.

**Figure 6:**
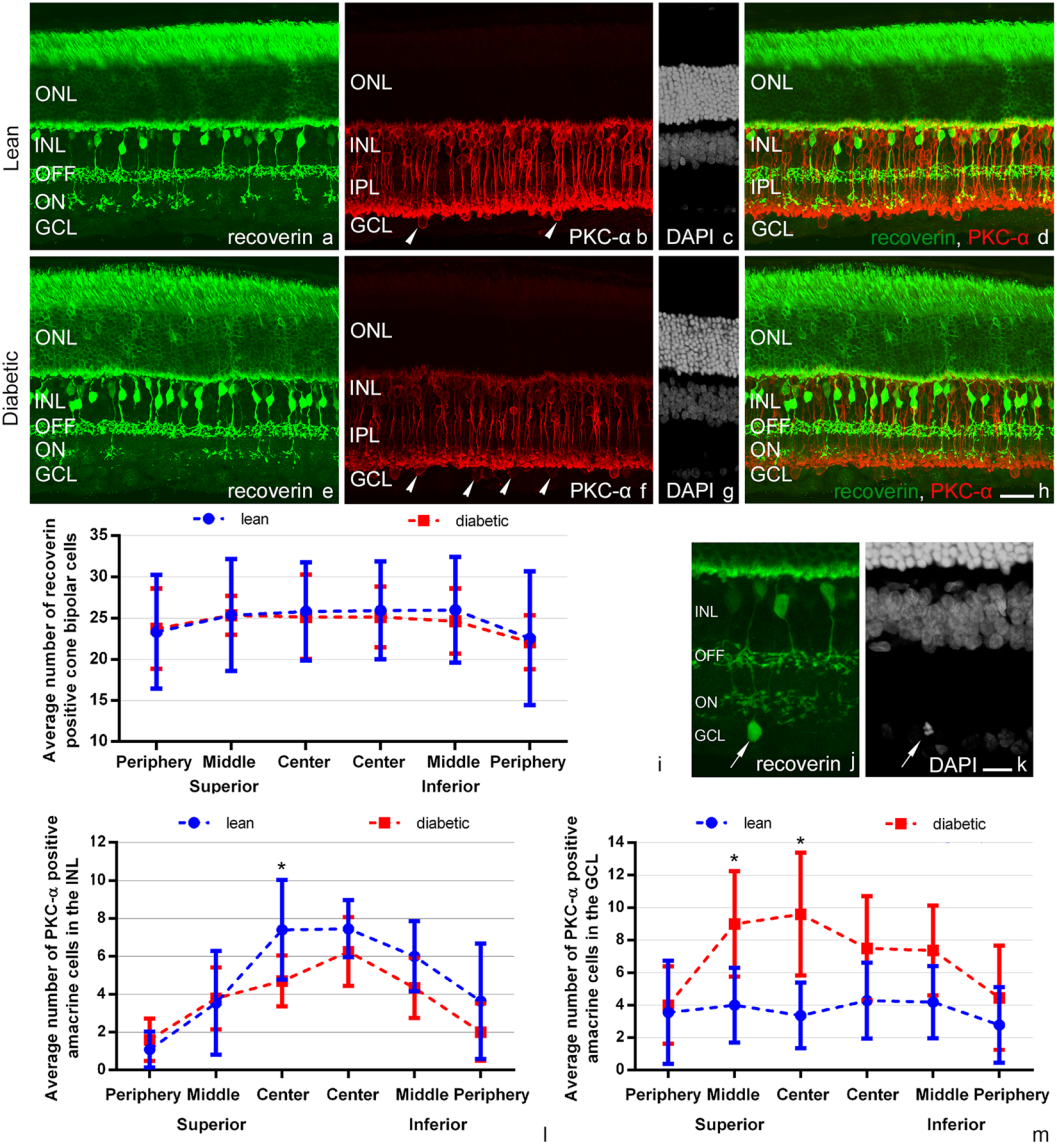
Cone and rod bipolar cells in diabetes. Cone and rod bipolar cells were analysed using anti-recoverin (**a**,**e**,**j** in green) and anti-PKC-α (**b**, **f** in red) antibodies, respectively. For orientation cell nuclei were labelled with DAPI-staining (**c**,**g**,**k**). Merged view: **d**, **h**. Both bipolar cell types including ON and OFF cone bipolar subtypes showed normal morphology in diabetes (second row) compared to controls (upper row). PKC-α also labelled at least one population of amacrine and displaced amacrine cells (arrowheads in **b** and **f**), while recoverin also stained all photoreceptor cells and a low number of cells in the ganglion cell layer with cone or rod like nuclear morphology (arrows in **j** and **k**). Quantitative analysis showed no evident change in the number of recoverin positive cells in any region of the retina (**i**). There was an increase in the number of PKC-α positive, displaced amacrine cells in the GCL in the superior central and mid-peripheral retinal regions (**m**). In contrast the number of PKC-α positive amacrine cells in the INL decreased or showed no change in diabetes (**l**). Significant differences (p < 0.05) were labelled with *. ONL: outer nuclear layer, INL: inner nuclear layer, IPL: inner plexiform layer, ON and OFF: sublayers of the IPL, GCL: ganglion cell layer. *Bar*: 20 μm (**a**–**h**) and 10 μm for **j** and **k**.

Rod morphology was examined with a polyclonal anti-rhodopsin (AO) antibody. Like in cones the morphology of rod outer segments seemed to be degenerated in almost all regions examined (Fig. 4b,d). Also in case of rods, checking single confocal planes from the stacks made it possible to decide if rod outer segments were degenerated or not - irrespectively whether they were aligned properly or obliquely - without any difficulty. Examples of single confocal plane images from healthy and degenerated outer segments are shown in Supplementary Fig. S6. Alternatively, rods could also be labelled with antibodies against rod phototransduction regulatory proteins: recoverin (Fig. 6a,e), rod arrestin (Fig. 5e,f) and rod transducin (Fig. 5g,h). In contrast to the altered morphology there was only a slight change in the distribution and staining intensity of regulatory proteins. In case of rod arrestin we found a detectable mislocalization of the protein to perikarya in diabetic specimens, restricted to the morphologically less preserved regions.

The possible changes in glycosylation of the interphotoreceptor matrix proteins were assessed with PNA (Fig. 5c,d) and WGA (Fig. 3c,g) lectins. Unlike in STZ-induced Sprague-Dawley T1D rats^13^, in this case no change was detectable.

### Retinal pigment epithelium

RPE-65 labels isomerohydrolase enzyme located in the pigmented epithelium. In a previous report^13^, visible change in RPE-65 activity along with a significant change in RPE thickness was reported in Wistar rats. In ZDF rats, although no precise quantification of thickness was attempted, a decrease in immunostaining was prominent (Fig. 4b,d).

### Bipolar cells

Besides the photoreceptors both the ON and OFF cone bipolar cells can be labelled with recoverin antibody^33^. Comparing diabetic and control retinal sections recoverin immunocytochemistry showed no major change in either the morphology (Fig. 6a,e) or the number (Fig. 6i) of these cell types stained. Details of the statistical analysis are given in Table S7. We also detected a minor recoverin-positive population amongst the ganglion cells with dendrites branching exclusively in the ON sublayer of the inner plexiform layer (IPL) (Fig. 6j,k). Literature and our own data indicate that these cells also contain rod or cone opsins^34, 35^. Although no precise quantification was attempted, these cells seem to be present with similar morphology and equally low numbers in diabetic specimen as well.

With PKC-α antibody we did not find any major qualitative difference in the rod bipolar cell population (Fig. 6b,f). Due to the overlapping cell bodies and their extremely high number, no quantification was attempted for rod bipolar cells.

### Changes in amacrine cells

Due to their diversity, amacrine cells can be detected with several different antibodies labelling various subpopulations.

Besides rod bipolar cells there was at least one amacrine and a displaced amacrine cell population also detectable with the PKC-α antibody (Fig. 6b,f). Regarding these two PKC-α positive amacrine cell populations we detected a significant increase in the number of displaced amacrine cells in the superior central and mid-peripheral regions in diabetic specimens compared to controls (Fig. 6m). A decrease in the number of PKC-α positive amacrine cells in the inner nuclear layer (INL) could also be observed in one region (Fig. 6l). Details of the statistics, with the appropriate p values are listed in Supplementary material, Tables S8 and S9.

**Figure 7:**
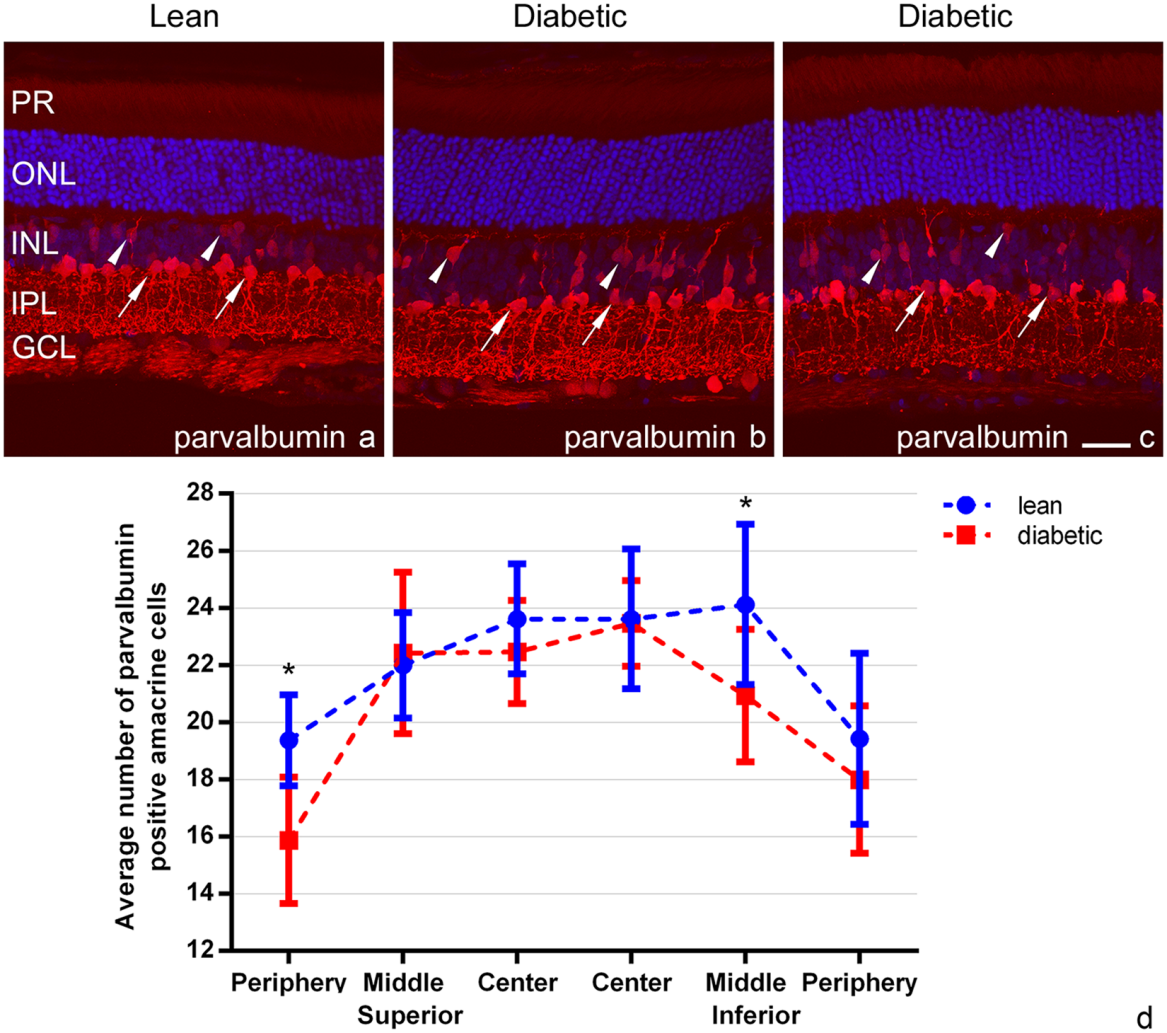
A decrease in the staining intensity and arborisation of parvalbumin stained AII amacrine cells. Parvalbumin (in red) stains mostly AII amacrine cells (arrows), a population of bipolar cells (arrowheads) and a few cells in the GCL. Compared to controls (**a**), there was a decrease in the staining intensity and arborisation of AII amacrine cells which was prominent in some regions (**c**), but less well visible in others (**b**). In most regions, there was no detectable change in the number of stained elements. Precise quantification (**d**) revealed a decrease only in the superior peripheral and inferior mid-peripheral regions (*: p < 0.05). DAPI is used as a nuclear staining on **a**, **b** and **c**. PR: photoreceptor layer, ONL: outer nuclear layer, INL: inner nuclear layer, IPL: inner plexiform layer, GCL: ganglion cell layer. *Bar*: 20 μm.

Parvalbumin stains dominantly AII amacrine cells, along with a few wide field amacrine cells, a population of bipolar cells and a few cells in the ganglion cell layer (GCL)^36^. We detected a moderate or markedly decreased staining intensity for all labelled cell types and incomplete arborisation in diabetic specimens (Fig. 7a–c). Beside the change in staining intensity, no difference in the number of labelled AII amacrine cells was detectable for the majority of the retina; except for the superior peripheral and inferior mid-peripheral counting frame, in which the decrease was significant (Fig. 7d). Details of the statistical analysis are given in Supplementary material, Table S10.

**Figure 8:**
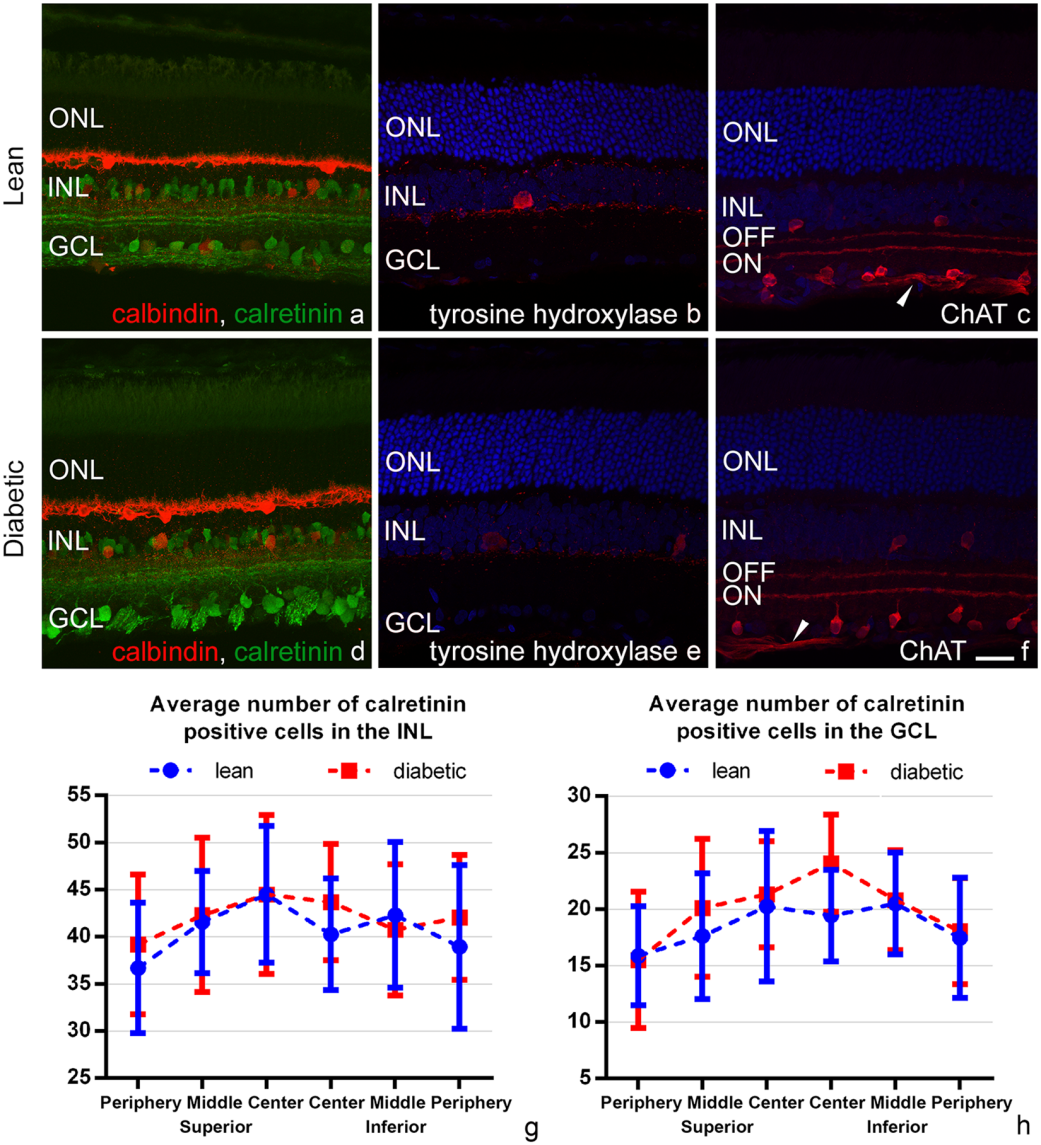
Staining patterns for calbindin, calretinin, tyrosine hydroxylase and choline acetyltransferase (ChAT) antibodies in lean (upper row) and diabetic rat retinas (lower row). Calbindin (**a**,**d**, in red) stains horizontal cells, and a few cells in the INL and GCL. Calretinin (**a**,**d**, in green) is a frequently used marker for some ganglion cells, amacrine and displaced amacrine cells, with processes terminating in three distinct layers of the IPL. No change in staining intensity or in the number of stained cells was evident for these two antibodies. Quantification for calretinin positive cells are given in **g** and **h**. Antibody against tyrosine hydroxylase (**b**, **e**, in red) labels dopaminergic amacrine cells. A slight decrease in staining intensity was detectable in diabetes (**e**). ChAT antibody (**c**,**f** in red) labels mostly cholinergic starburst amacrine cells, along with some axons in the nerve fibre layer (arrowheads) and possibly some ganglion cells also. No change was evident in diabetes. DAPI is used as a nuclear staining. ONL: outer nuclear layer, INL: inner nuclear layer, GCL: ganglion cell layer, ON and OFF: sublayers of the IPL. *Bar*: 20 μm.

Calretinin is a frequently used marker staining mostly amacrine and displaced amacrine cells with processes terminating in three distinct layers of the IPL. A few ganglion cells can also be labelled^37^. Comparing control and diabetic retinas, no difference was evident in staining intensity (Fig. 8a,d) and in the number of stained elements located in the INL or GCL (Fig. 8g,h). Details of the statistical analysis, with the appropriate p values are listed in Tables S11 and S12. Only a slight disorganization of the IPL sublayers was detectable (Fig. 8d) in diabetic specimens, however it was not a consistent finding and was restricted to a few retinal regions only.

Tyrosine hydroxylase labels dopaminergic amacrine cells^38^. A prominent decrease in staining intensity was detectable in diabetic rats (Fig. 8b,e), furthermore there was a significant change in the number of stained elements (7.9 ± 1.7 labelled cell bodies per section in control and 6.3 ± 2.2 in diabetes, p: 0.045).

Choline acetyltransferase labels two populations of starburst amacrine cells with processes terminating in the ON and OFF sublamina of the IPL, respectively. Although no precise quantifications were performed, diabetes does not seem to cause any major qualitative and quantitative change in their distributions (Fig. 8c,f).

### Horizontal cells

The antibody against calbindin stains horizontal cells and a few cells in the INL and GCL under control conditions^36^. Comparing sections of diabetic and lean specimens (Fig. 8a,d), we did not find any qualitative or significant quantitative changes in calbindin labelling. The number of horizontal cells per section was 116.3 ± 13.9 in lean vs. 125.7 ± 14.8 in diabetic specimens (p: 0.06).

### Ganglion cells

**Figure 9:**
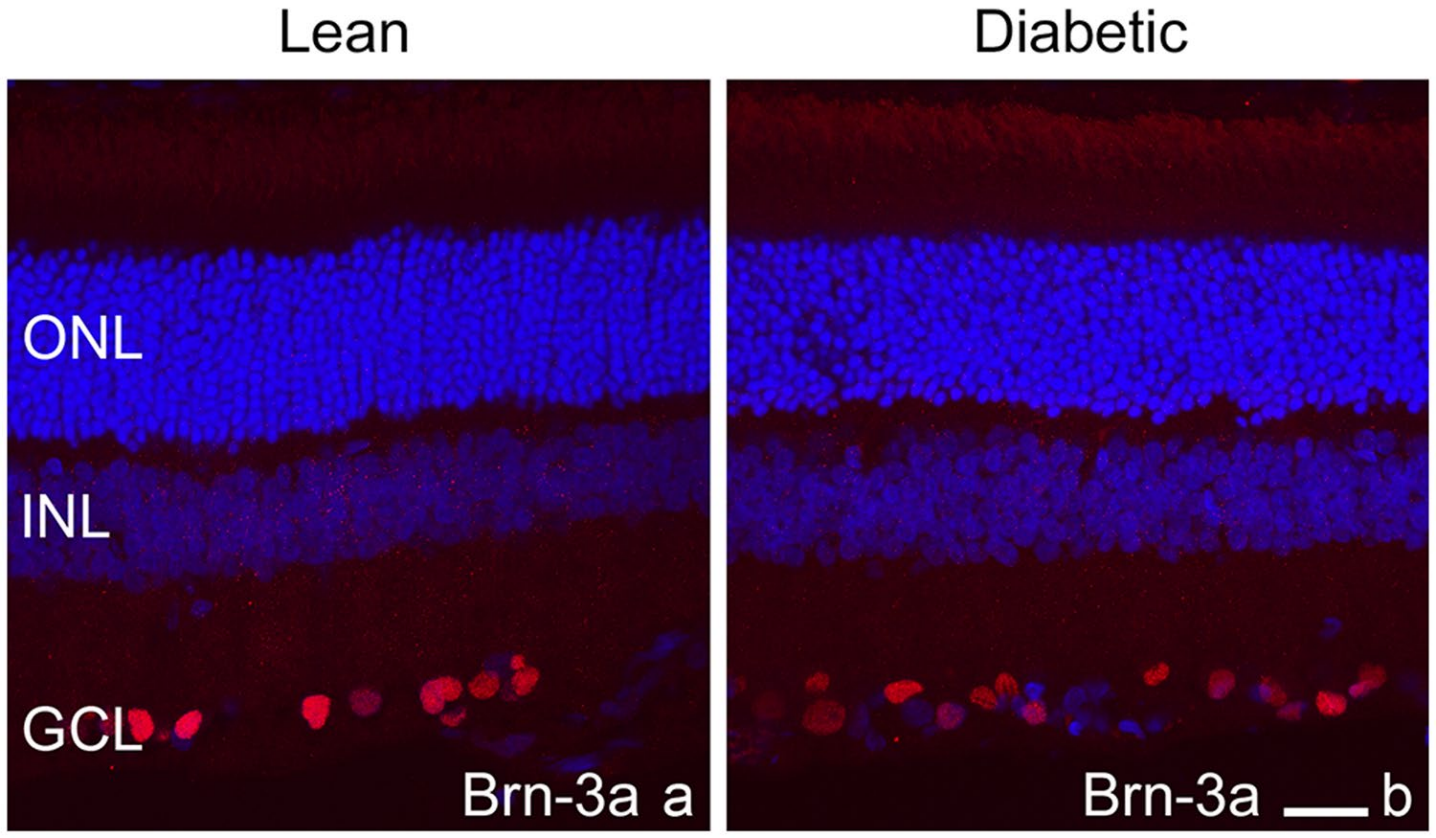
There was no major change in ganglion cell staining detected by Brn-3a antibody in control (**a**) and diabetic (**b**) specimens. DAPI is used as a nuclear staining. ONL: outer nuclear layer, INL: inner nuclear layer, GCL: ganglion cell layer. *Bar*: 20 μm.

Many markers used in our study labelled a smaller or larger population of ganglion cells including recoverin, calretinin, parvalbumin, cone arrestin, rod arrestin and rod transducin. Although no precise counting was performed specifically for the ganglion cells with most of the antibodies, no noticeable difference was evident. In order to estimate the possibility and the rate of overall ganglion cell loss, we used an antibody against Brn-3a (Fig. 9a,b). Literature data indicate that the majority of ganglion cells can be labelled with this antibody^39^. We calculated the total number of labelled cells per sections comparing diabetic and control specimens (n = 4 sections from each retina, from 4 specimens from each group) and observed no significant difference (319.4 ± 49.8 cells per section in lean vs. 346.5 ± 51.2 cells per section in diabetic specimens, p: 0.23).

## Discussion

Although T2D rat models such as ZDF, OLETF, SDT rats etc. were established decades ago (reviews:^16, 40^), ocular lesions were addressed on these models only by few reports, in which mostly the pathology of retinal capillaries was studied in details. In 6–7 months old ZDF rats, retinal capillaries were reported to be hypercellular and basement membranes thicker than in lean rats of the same age^41^. Elevated expression of inflammatory cytokines, oxidative stress markers and VEGF were also demonstrated in 14 weeks old ZDF rats^42^, while the gene expression of these factors were studied at 32 weeks of age^43^. Ischemic features of the human diabetic retina like acellular capillaries and pericyte loss were absent in 6-7 months old ZDF rats^40^ but appeared at later stages examined (33–34 postnatal weeks:^44^; 32 postnatal weeks:^43^). Apart from the capillary structure, histopathological data on ZDF rat retina in literature are extremely sparse. There are reports on Müller glia activation^45^, increased rate of apoptosis already in 19 weeks old ZDF rats^46^ or contradictorily on no change in retinal thickness and apoptosis rate even at 23 weeks of age^45^.

To fill this gap, in this report we gave a detailed immunocytochemical analysis on the histopathology of both glial and neural cell types of ZDF rat retinas. We used the same palette of antibodies that we had utilized previously on STZ-induced diabetic models^13, 14^ in order to compare T1D and T2D directly with the same methodology and to draw possible conclusions about the factors contributing to the alterations detected. Numerous metabolic differences exist between the early stages of type 1 and type 2 diabetes (e.g. opposite changes in insulin levels, insulin resistance, obesity, hyperlipidaemia etc. – ref. ^47^) that may all have effect on retinal histopathology. Therefore our original assumption was that T2D rat models would show differences in the type of retinal cells affected or in the rate of damage.

However, the data presented here reveal a surprising similarity of the pathology of T1D and T2D rat models examined. Both models feature a prominent glia activation, outer segment degeneration for almost all M-cones and rods, changes in cone-opsin expression patterns, a decreased expression of RPE-65 in pigment epithelial cells and changes in the labelling patterns and the morphology of some amacrine cell types. We can thus conclude that despite the different evolution of the two types of the disease, the neuroretinal cells effected are the same in both subtypes of diabetes.

Furthermore, Müller glia activation with a prominent upregulation of GFAP expression was accompanied by a downregulation (T1D – ref. ^14^) or unaltered (ZDF) expression of vimentin intermediate filaments. This opposite alteration pattern seems to be specific for diabetic conditions^14^. In most other pathologies both GFAP and vimentin is upregulated^48^. Under cell culture conditions similar expression pattern can be detected experimentally, if Müller glia cells are co-cultured with activated microglia cells^49^. So in this study we specifically examined the microglia response in diabetes and detected an increase in their number – a situation mimicking the published culture conditions. Whether this unusual Müller glia activation has any specific role in the pathophysiology of the disease requires further investigations.

Considering the metabolic differences between the early stages of T1D and T2D (e.g. opposite changes in insulin levels, insulin resistance, obesity, hyperlipidaemia^47^) the similarity of their neuroretinal pathology is rather surprising, with special respect to the near equal rate of photoreceptor outer segment degeneration in both models. Although in our previous work^13^ outer segment degeneration of rods was also confirmed by electron microscopy, here – due to the complex nature of the study – sacrificing sections for this purpose was not possible. However, the relatively thick sections prepared for confocal microscopy, allowed us to follow individual outer segments and confirm degeneration on the stacks or on single confocal planes. This technique was especially useful in case of cones, for which outer segment degeneration is almost impossible to determine on electron microscopic sections, due to their low number in rodents (cones comprise less than 1% of all photoreceptors in the rat retina – refs 30 and 31).

It has been long proposed that insulin is one of the key factors in the development and surviving of the cone and rod outer segment^23, 24^. Yet, despite the initially opposite changes in insulin levels, no major difference was evident in morphology (at least) in the diabetic stage examined. This can be explained by two distinct propositions. On the one hand rats were insulinopenic approximately from postnatal week 20^18^ - for about 12 weeks in our experiment - and this relatively long period might have been enough to diminish the possible protective effect (if any) of early elevated insulin levels. Alternatively, hyperglycaemia alone might be sufficient to induce most pathologic alterations of the disease, while altered insulin signalling plays a less decisive role. Hyperglycaemia has also been suggested by others as a major casual factor in diabetic retinal and neural degeneration that may contribute to the development of pathological alterations through several possible mechanisms, like polyol pathway, oxidative stress, non-enzymatic glycation, oedema formation etc. (For review see refs 27 and 50).

To develop diabetes in animals, some kind of chemical induction (e.g. streptozotocin) is a technique often applied. When studying diabetic models, especially the neural effects of diabetes, one should always consider that not only the metabolic changes but also the chemical itself used for the induction may give rise to some of the neural alterations^51^. Our results presented here, with histological pictures similar to T1D models^13, 14^, exclude any major role of STZ in the development of the detected pathologies.

There are few minor and mostly quantitative differences detected by us between the animal models of T1D and T2D which may be due to the different duration of hyperglycaemia. In our study, STZ-induced rats were analysed three months after the induction, with continuously high glucose levels (>20 mmol/l) throughout the observation period. In ZDF rats blood levels were in the same range for approximately five months (from 12^th^ to 32^nd^ weeks), with moderately high values between the 7^th^ and 12^th^ weeks. In STZ-induced diabetes, we detected a patchy appearance of Müller glia activation and AII amacrine cell degeneration. In contrast, in ZDF rats Müller glia cells were activated all over the retina, while parvalbumin showed a highly-degenerated profile for the majority of the retina. Also, whereas no decrease in numbers could be confirmed for any cell types counted in the STZ-induced model, in ZDF we detected a small but already significant decrease in the number of some of the elements counted (AII amacrine cells, dopaminergic amacrine cells), sometimes restricted to some retinal regions. As we proposed earlier^14^ the initially patchy degeneration later may appear all over the retina with the progression of the disease and finally the degenerated cells begin to be eliminated. The results presented here are certainly in line with this proposition.

There are a number of additional factors known to occur in diabetes, like impaired autoregulation^52^ oxidative stress^53–55^ and inflammatory citokine expression^42, 43^ – that were not examined in detail in the study. We can not rule out therefore, that these factors also contribute to the development of the detected alterations.

Apoptosis is one of the earliest and most often documented features of diabetic retinopathy, present in human diabetic patients^56^ as well as in practically all animal models^44, 57, 58^. Apoptotic cells were detectable related to the vasculature^44^ and amongst neural elements^56^ eventually leading to the thinning of the neural retinal layers^57^. We must emphasize that despite a slight but already detectable decrease in the number of certain cell populations, at this stage examined there was no major loss of cells as proven clearly by the cell counts, and the lack of significant increase in the number of apoptotic bodies or retinal thickness. This is in agreement with the results on 23 weeks old animals of Johnson *et al*.^45^. but contrary to other observations^46^ reporting an increase in the number of apoptotic cells already in 19 weeks old ZDF rats. The reason for this discrepancy is currently unknown but may be related to the differences in animal care, number of sections examined and reagents used. The lack of detectable increase in TUNEL-positive elements and reduction in retinal thickness clearly indicate that the histological alterations presented here in ZDF rats also precede significant retinal cell loss – as well as in STZ-induced diabetic rats^13, 14^.

In our previous reports on T1D rat model^13, 14^ we concluded that the histological alterations described by us may be related to some of the early detectable symptoms of diabetic patients, like ERG changes and colour vision defects^5, 59^. In our present study, we also found similar signs of degeneration in the rod pathway, like rod outer segment degeneration, rod arrestin mislocalization and changes in the morphology and number of AII amacrine cells that - in theory - may all contribute to the alteration of scotopic ERG reported in diabetic patients^59, 60^ and in animal models^45, 60^. Colour vision defects^61, 62^ and photopic ERG changes^63^ can be related to changes in cone opsin expression pattern and M-cone outer segment degeneration. Changes in oscillatory potentials^45^ may be the ERG manifestations of amacrine cell dysfunctions – from which AII amacrine cells, dopaminergic amacrine cells and PKC-α positive amacrine cells certainly show alterations in ZDF rats, as well as STZ induced diabetic rats. The relationship between histopathology described by us and functional alteration thus seems to be a reasonable conclusion but at this stage, we lack direct evidence.

Available ERG data from ZDF rats in literature are extremely sparse. Data published from 8 to 22 weeks old animals show an increase in a-wave amplitude and maximum slope under scotopic conditions^45^. These changes are different from those in human patients and type 1 diabetic rats. Similarly to other models and human data however they show delay in oscillatory potentials^45^. No electrophysiological data are available in literature from later stages that could correspond to our study. Further studies are required to obtain a more detailed picture about the possible connection between functional changes and histological alterations.

In summary, we report the first detailed description on the histopathology of the neural retina of the ZDF rats, one of the most commonly used T2D model. We present alterations similar to those detected in STZ-induced T1D rats and conclude that the cell types effected are the same in both types of the disease, and hypothesize that the results presented could underlie the functional alterations detected in human patients and animal models. These findings support the idea that both rat models are beneficial for studying early diabetic alterations of the neuroretina.

## Materials and Methods

### Animal handling

All procedures of the present study were performed in concordance with the Association for Research in Vision and Ophthalmology (ARVO) statement for the Use of Animals in Ophthalmic and Vision Research and were approved by the local Ethical Committee for Animal Experimentation of the Semmelweis University and by the Animal Health and Animal Welfare Directorate of the National Food Chain Safety Office of the Hungarian State (number of approval: 22.1/1162/3/2010).

Experiments were carried out on ZDF inbred rats, where T2D and related complications develop due to a leptin receptor gene mutation and a special diet. Homozygous recessive males (*fa/fa*) develop obesity, fasting hyperglycaemia and T2D. Homozygous dominant (+/+) and heterozygous (*fa*/+) genotypes remain normoglycemic (ZDF lean). ZDF rats (n = 8) and ZDF lean controls (n = 8) were obtained from Charles River Laboratories (Sulzfeld, Germany) at the age of 6 weeks, housed in a room with constant temperature (22 ± 2 °C) under a 12-12 h alternating light-dark cycle. Rats were supplied with a special diet (Purina 5008 as recommended by the supplier) and water ad libitum. Blood glucose levels were checked at the 7^th^ week, and every five weeks thereafter. Body weights were measured prior to anaesthesia. At the age of 32 weeks, anaesthesia was induced and maintained with isoflurane (3–5% and 1.5–3%, respectively). A drop of blood was collected from the tail vein for blood glucose determination carried out with a digital glucose meter and test strips (Accu-Chek^®^ Sensor, Roche Inc., Mannheim, Germany). Rats were tracheotomized, intubated, artificially ventilated and invasive hemodynamic measurements were carried out using a Millar microcatheter (Millar Inc, Huston, Texas, USA), as described elsewhere^64^. Detailed results of the hemodynamic measurements will be given in another report.

To remove erythrocytes from tissues an *in vivo* perfusion was performed. After opening the thoracic cavity and dissecting the inferior vena cava, a total volume of 40 ml oxygenated Ringer solution (37 °C) was infused into the left ventricle through the apex of the heart with a speed of 8 ml/min. All animals were euthanized by exsanguination and decapitated. The eyes were removed and placed into fixative within 10 minutes after euthanasia.

### Tissue preparation and immunohistochemistry

Oriented and enucleated eyes were cut at the ora serrata, then the cornea, lens and vitreous body were removed, finally the posterior eyecups were immersed in freshly prepared fixative solution (4% paraformaldehyde diluted in 0.1 M phosphate buffer [PB, pH 7.4]), for two hours at room temperature (RT). After extensive rinsing in 0.1 M PB, cryoprotection (in 30% sucrose diluted in 0.1 M PB) was applied overnight at 4 °C, then the eyecups were embedded in tissue-embedding medium (Shandon Cryomatrix, Thermo Scientific, UK). 20 μm thick cryosections were cut vertically and stored at −20 °C until use.

Immunohistochemical procedures were carried out on cryosections according to protocols previously published by our laboratory^35^. In brief, prior to incubation with primary antibodies, to block nonspecific staining, sections were treated with 1% bovine serum albumin diluted in 0.1 M phosphate buffered saline (PBS, pH 7.4) with additional 0.4% Triton X-100 (Sigma-Aldrich Kft, Budapest, Hungary) for 2 hours at RT. Primary antibodies (details are listed in Table 1) were applied on sections overnight at 4 °C. After repeated rinsing, species-specific fluorescent probes (Alexa 488 or Alexa 594 conjugates, 1:200, Life Technologies, Carlsbad, CA) were applied for 2 hours at RT. Cell nuclei were counterstained with DAPI (4,6-diamidino-2-phenylindole, Sigma-Aldrich Kft, Budapest, Hungary). Sections with the primary antibodies omitted were used as negative controls. Most of the antibodies applied have been tested and validated in rat retinas previously^13, 14, 35^.

**Table 1:** Primary antibodies used in the study.

Antibodies (name, clone and cat. number)	Source	Working concentration	Host and type	Epitope specificity or labelling pattern in rats	Reference
glial fibrillary acidic protein (GFAP), Clone:G-A-5, #G-3893	Sigma-Aldrich Kft., Budapest, Hungary	1:1000	mouse monoclonal	astrocytes and Müller cells in retinal injuries	^7^
vimentin, Clone:V-9, #MAB3400	Millipore, Billerica, MA, USA	1:10000	mouse monoclonal	Müller cells	^28^
Iba1, #019-19741	Wako Chemicals Inc, USA	1:500	rabbit polyclonal	microglia	^67^
Rb X opsin red/green, #AB5405	Millipore, Billerica, MA	1:1000	rabbit polyclonal	M-cone opsin	^68^
anti-opsin blue, #AB5407	Millipore, Billerica, MA	1:1000	rabbit polyclonal	S-cone opsin	^69^
COS-1	produced in our laboratory	1:50	mouse monoclonal	M-cone opsin	^70^
OS-2	produced in our laboratory	1:5000	mouse monoclonal	S-cone opsin	^70^
cone arrestin, #AB15282	Millipore, Billerica, MA	1:1000	rabbit polyclonal	cone photoreceptors	^71^
rhodopsin (AO)	produced in our laboratory	1:1000	rat polyclonal	rod photoreceptors	^70^
recoverin	kind gift of Karl-Wilhelm Koch, University of Oldenburg, Germany	1:500	rabbit polyclonal	cone bipolar cells, rod and cone photoreceptors, few cells in GCL	^33^
rod arrestin	generous gift of Igal Gery, National Eye Institute, Bethesda, MD, USA	1:400	rabbit polyclonal	rod photoreceptors	^72^
rod transducin, #AB74059	Abcam, Cambridge, UK	1:300	rabbit polyclonal	rod photoreceptors	^73^
retinal pigment epithelium-specific 65 kDa protein (RPE-65), #MAB5428	Merck Kft., Budapest, Hungary	1:500	mouse monoclonal	isomerohydrolase (retinal pigment epithelium)	^74^
protein kinase c-alpha (PKC-α), #SC-8393	Santa Cruz Biotechnology, Santa Cruz, CA, USA	1:200	mouse monoclonal	rod bipolar cells, few amacrine and displaced amacrine cells	^75^
parvalbumin, Clone:PARV-19, #P-3088	Sigma-Aldrich Kft., Budapest, Hungary	1:300	mouse monoclonal	AII and some widefield amacrine cells, a small number of bipolar and ganglion cells	^36^
calretinin, #AB5054	Millipore, Billerica, MA, USA	1:2500	polyclonal rabbit	amacrine cells in the IPL, amacrine and ganglion cells in the GCL	^37, 76^
tyrosine hydroxylase (TH), #MAB5280	Millipore, Billerica, MA, USA	1:250	mouse monoclonal	dopaminergic amacrine cells	^38^
choline acetyltransferase (ChAT), #AB144P	Millipore, Billerica, MA, USA	1:200	goat polyclonal	cholinergic amacrine cells	^77^
calbindin D28k, #300	Swant, Marly, Switzerland	1:200	mouse monoclonal	horizontal cells, few amacrine cells, few cells in GCL	^36, 78^
Brn-3a, #SC-31984	Santa Cruz Biotechnology, Inc., Heidelberg, Germany	1:500	goat polyclonal	ganglion cells	^79^

Lectin histochemistry was also applied on a few sections. List of the lectins used, with the staining characteristics are given in Table 2. Lectins were either used in a biotinylated or fluorescent isothiocyanate (FITC, in case of isolectin B_4_) conjugated form (2 h, RT) and were detected with streptavidin linked Alexa dies, or Alexa 488 conjugated anti-FITC antibody (1:200, Life Technologies, Carlsbad, CA).

**Table 2:** Tested lectins and their labelling pattern in the retina.

Lectin	Source	Working concentration	Ligand motif	Labelling pattern	Reference
lectin from *Triticum vulgaris* (wheat germ agglutinin lectin - WGA)	Vector Laboratories Inc., Burlingame, CA	50 µg/ml	N-acetyl-D-glucosamine and sialic acid	interphotoreceptor matrix of rods, microglia cells	^80, 81^
lectin from *Bandeiraea simplicifolia (Griffonia simplicifolia)* - (Isolectin B_4_)	Sigma-Aldrich Kft., Budapest, Hungary	20 µg/ml	α-D-galactosyl-residues	microglia cells, blood vessels, some cones	^82^
lectin from *Arachis hypogaea* (peanut agglutinin lectin -PNA)	Sigma-Aldrich Kft., Budapest, Hungary	5 µg/ml	galactosyl-β(1,3)- N-acetyl-D-galactosamine	interphotoreceptor matrix of cones, IPL sublayers	^83, 84^

### Measurement of retinal thickness

To assess the effect of diabetes on the thickness of the retina, the distance between the outer and inner limiting membranes (ILM-OLM), and the thickness of the outer nuclear layer (ONL) alone was measured at six given locations (250 and 500 μm (central values), as well as 4000 μm (peripheral values) from the optic nerve head in both superior and inferior directions), on four sections per each retina derived from four specimens from each group.

In the ONL, nuclei are typically arranged in columns on well oriented sections. To further estimate the number of photoreceptor cells, additionally we also counted the number of nuclei in the ONL in at least three columns per each location parallel with the thickness measurements.

Only vertical cryosections at the level of the optic nerve were analysed using a Zeiss Axiophot (Carl Zeiss, Oberkochen, Germany) microscope with a 40x (NA: 1,4) oil immersion objective.

### Cell counting

Unless otherwise indicated cell counting was performed on 20 µm thick vertical sections (n = 4 sections per specimen) taken from both diabetic (n = 4) and lean (n = 4) rats. For cell counting only the sections going through the optic nerve head were selected, sections taken from the same specimen were approximately 100 µm away from each other in the temporo-nasal direction.

In case of horizontal cells (labelled with calbindin), dopaminergic amacrine cells (labelled with TH), microglia cells (Iba1) and ganglion cells (Brn-3a) the total number of the stained cells per sections were counted. For counting M-cones, calretinin, parvalbumin and recoverin positive elements 350 µm long central (immediately adjacent to the optic nerve head), mid-peripheral (approximately 3 fields – 875 µm away from the optic disc) and peripheral (approximately 6 fields – 1925 µm away from the optic nerve) regions were chosen in both superior and inferior directions. Calretinin positive cells were counted separately in the INL and GCL.

### Apoptosis

To define the number of apoptotic cells terminal deoxynucleotidyl transferase deoxyuridine triphosphate nick end labelling (TUNEL, *In situ* Cell Death Detection Kit, Fluorescein; Roche Diagnostics, Mannheim, Germany) assay was used on complete vertical sections through the optic nerve of both diabetic (n = 4) and lean (n = 4) animals with 4 sections per each specimen. For negative controls, we have used sections incubated omitting the terminal transferase enzyme, while positive control sections were pre-incubated with DNAse I prior to performing the TUNEL reaction.

### Statistical analysis

All data were represented as mean ± standard deviation (SD). Statistical analysis was performed using R Statistical Program^65^. In case of body weight measurements, and counting of horizontal cells (labelled with calbindin), dopaminergic amacrine cells (labelled with TH), microglia cells (Iba1) and ganglion cells (Brn-3a) the normality of the data was tested with the Shapiro-Wilk’s test, while for comparing the deviations the F-test was used. To investigate the possible difference between cell numbers in the lean and diabetic groups, in case of normal distribution of data two-sample Student’s t-test for equal deviation and Welch test for unequal deviations was performed. Non-parametric data were analysed with Mann-Whitney test. P-values less than 0.05 were considered significant.

For analysing blood glucose levels, two-way repeated measures ANOVA test, while for the statistical analysis of retinal- and ONL thickness, number of nuclei in the ONL, calretinin, parvalbumin and recoverin positive elements two-way ANOVA test was used with the Bonferroni post hoc test. P-values less than 0.05 were considered significant.

### Imaging

Sections were viewed and images were recorded with a Zeiss LSM 780 Confocal System coupled to a Zeiss Axio Imager upright microscope, using Zen 2012 software (Carl Zeiss, Oberkochen, Germany) with identical setting for diabetic and lean specimens. Representative images were reconstructed with ImageJ software^66^ using information of adjacent Z-stacks from 10 µm section thickness projected with maximum intensity projection. The final montages were created and labelling was added by Adobe Photoshop 7.0 (San Diego, CA, USA). Only minor adjustments concerning brightness and contrast were made parallel on some picture pairs (Fig. 3 insets, d,h; Figs 4e–h, 5a,b and 8b,e) to enhance the visibility of the morphologic features demonstrated.

## Electronic supplementary material

Supplementary Information
