# Supplementary material for: Histological Evaluation of Diabetic Neurodegeneration in the Retina of Zucker Diabetic Fatty (ZDF) Rats
Source: Sci Rep. 2017 Aug 21;7:8891. doi: 10.1038/s41598-017-09068-6 (PMC5566374; doi:10.1038/s41598-017-09068-6)
Supplement: Supplementary file 1 — Supplementary Information [file 41598_2017_9068_MOESM1_ESM.pdf]

# Supplementary Information

# Histological Evaluation of Diabetic Neurodegeneration in the Retina of Zucker Diabetic Fatty (ZDF) Rats

Klaudia Szabó[1], Anna Énzsöly[1,2], Bulcsú Dékány[1], Arnold Szabó[1], Rozina I. Hajdú[1], Tamás Radovits[3], Csaba Mátyás[3], Attila Oláh[3], Lenke K. Laurik[2], Gábor M. Somfai[2], Béla Merkely[3], Ágoston Szél[1], Ákos Lukáts[1*]

[1] Department of Anatomy, Histology and Embryology, Semmelweis University, Budapest, H-1085, Hungary

[2] Department of Ophthalmology, Semmelweis University, Budapest, H-1085, Hungary

[3] Heart and Vascular Center, Semmelweis University, Budapest, H-1085, Hungary

* Corresponding author:

E-mail: lukatsakos@gmail.com (LÁ)

**Table S1. Supplementary data to the statistical analysis of blood glucose levels**

| | Lean | | Diabetic | | Lean - Diabetic | | |
|---|---|---|---|---|---|---|---|
| | Mean | SD | Mean | SD | Significant? | Summary | Adjusted P Value |
| 7th week | 5,7 | 0,6324555 | 8,357142 | 1,263404 | Yes | * | 0,0203 |
| 12th week | 5,925 | 0,9300538 | 21,74286 | 3,313536 | Yes | **** | < 0,0001 |
| 18th week | 5,3375 | 0,266927 | 27,37143 | 1,803436 | Yes | **** | < 0,0001 |
| 23rd week | 5,375 | 0,5750775 | 27,42857 | 2,138313 | Yes | **** | < 0,0001 |
| 28th week | 5,2875 | 0,8374067 | 20,94286 | 1,729024 | Yes | **** | < 0,0001 |
| 32nd week | 10,15 | 1,73617 | 27,87143 | 2,796852 | Yes | **** | < 0,0001 |

**Comparism between different postnatal ages**

**Lean**

| | Significant? | Summary | Adjusted P Value |
|---|---|---|---|
| 7th week vs. 12th week | No | ns | > 0,9999 |
| 7th week vs. 18th week | No | ns | > 0,9999 |
| 7th week vs. 23rd week | No | ns | > 0,9999 |
| 7th week vs. 28th week | No | ns | > 0,9999 |
| 7th week vs. 32nd week | Yes | **** | < 0,0001 |
| 12th week vs. 18th week | No | ns | > 0,9999 |
| 12th week vs. 23rd week | No | ns | > 0,9999 |
| 12th week vs. 28th week | No | ns | > 0,9999 |
| 12th week vs. 32nd week | Yes | **** | < 0,0001 |
| 18th week vs. 23rd week | No | ns | > 0,9999 |
| 18th week vs. 28th week | No | ns | > 0,9999 |
| 18th week vs. 32nd week | Yes | **** | < 0,0001 |
| 23rd week vs. 28th week | No | ns | > 0,9999 |
| 23rd week vs. 32nd week | Yes | **** | < 0,0001 |
| 28th week vs. 32nd week | Yes | **** | < 0,0001 |

**diabetic**

| | Significant? | Summary | Adjusted P Value |
|---|---|---|---|
| 7th week vs. 12th week | Yes | **** | < 0,0001 |
| 7th week vs. 18th week | Yes | **** | < 0,0001 |
| 7th week vs. 23rd week | Yes | **** | < 0,0001 |
| 7th week vs. 28th week | Yes | **** | < 0,0001 |
| 7th week vs. 32nd week | Yes | **** | < 0,0001 |
| 12th week vs. 18th week | Yes | **** | < 0,0001 |
| 12th week vs. 23rd week | Yes | **** | < 0,0001 |
| 12th week vs. 28th week | No | ns | > 0,9999 |
| 12th week vs. 32nd week | Yes | **** | < 0,0001 |
| 18th week vs. 23rd week | No | ns | > 0,9999 |
| 18th week vs. 28th week | Yes | **** | < 0,0001 |
| 18th week vs. 32nd week | No | ns | > 0,9999 |
| 23rd week vs. 28th week | Yes | **** | < 0,0001 |
| 23rd week vs. 32nd week | No | ns | > 0,9999 |
| 28th week vs. 32nd week | Yes | **** | < 0,0001 |

**Table S2. Supplementary data to the statistical analysis of retinal thickness**

| | Lean | | Diabetic | | Lean - Diabetic | | |
|---|---|---|---|---|---|---|---|
| | Mean | SD | Mean | SD | Significant? | Summary | Adjusted P Value |
| Superior 4000 µm | 100,8111 | 7,389884 | 113,7411 | 5,394616 | Yes | * | 0,0229 |
| Superior 500 µm | 158,3611 | 6,124254 | 180,3833 | 20,92404 | Yes | **** | < 0,0001 |
| Superior 250 µm | 169,9778 | 3,897598 | 181,7289 | 15,01863 | Yes | * | 0,0498 |
| Inferior 250 µm | 166,65 | 8,402864 | 186,8278 | 3,806479 | Yes | **** | < 0,0001 |
| Inferior 500 µm | 155,1556 | 7,230998 | 174,9411 | 4,851515 | Yes | **** | < 0,0001 |
| Inferior 4000 µm | 88,82111 | 3,425016 | 107,5078 | 7,355428 | Yes | *** | 0,0003 |

**Comparism between different retinal positions**

**Lean**

| | Significant? | Summary | Adjusted P Value |
|---|---|---|---|
| Sup. 4000 µm vs. Sup. 500 µm | Yes | **** | < 0,0001 |
| Sup. 4000 µm vs. Sup. 250 µm | Yes | **** | < 0,0001 |
| Sup. 4000 µm vs. Inf. 250 µm | Yes | **** | < 0,0001 |
| Sup. 4000 µm vs. Inf. 500 µm | Yes | **** | < 0,0001 |
| Sup. 4000 µm vs. Inf. 4000 µm | No | ns | 0,1068 |
| Sup. 500 µm vs. Sup. 250 µm | No | ns | 0,1357 |
| Sup. 500 µm vs. Inf. 250 µm | No | ns | 0,9043 |
| Sup. 500 µm vs. Inf. 500 µm | No | ns | > 0,9999 |
| Sup. 500 µm vs. Inf. 4000 µm | Yes | **** | < 0,0001 |
| Sup. 250 µm vs. Inf. 250 µm | No | ns | > 0,9999 |
| Sup. 250 µm vs. Inf. 500 µm | Yes | * | 0,0148 |
| Sup. 250 µm vs. Inf. 4000 µm | Yes | **** | < 0,0001 |
| Inf. 250 µm vs. Inf. 500 µm | No | ns | 0,1466 |
| Inf. 250 µm vs. Inf. 4000 µm | Yes | **** | < 0,0001 |
| Inf. 500 µm vs. Inf. 4000 µm | Yes | **** | < 0,0001 |

**diabetic**

| | Significant? | Summary | Adjusted P Value |
|---|---|---|---|
| Sup. 4000 µm vs. Sup. 500 µm | Yes | **** | < 0,0001 |
| Sup. 4000 µm vs. Sup. 250 µm | Yes | **** | < 0,0001 |
| Sup. 4000 µm vs. Inf. 250 µm | Yes | **** | < 0,0001 |
| Sup. 4000 µm vs. Inf. 500 µm | Yes | **** | < 0,0001 |
| Sup. 4000 µm vs. Inf. 4000 µm | No | ns | > 0,9999 |
| Sup. 500 µm vs. Sup. 250 µm | No | ns | > 0,9999 |
| Sup. 500 µm vs. Inf. 250 µm | No | ns | > 0,9999 |
| Sup. 500 µm vs. Inf. 500 µm | No | ns | > 0,9999 |
| Sup. 500 µm vs. Inf. 4000 µm | Yes | **** | < 0,0001 |
| Sup. 250 µm vs. Inf. 250 µm | No | ns | > 0,9999 |
| Sup. 250 µm vs. Inf. 500 µm | No | ns | > 0,9999 |
| Sup. 250 µm vs. Inf. 4000 µm | Yes | **** | < 0,0001 |
| Inf. 250 µm vs. Inf. 500 µm | No | ns | 0,1142 |
| Inf. 250 µm vs. Inf. 4000 µm | Yes | **** | < 0,0001 |
| Inf. 500 µm vs. Inf. 4000 µm | Yes | **** | < 0,0001 |

**Table S3. Supplementary data to the statistical analysis of ONL thickness**

| | Lean | | Diabetic | | Lean - Diabetic | | |
|---|---|---|---|---|---|---|---|
| | Mean | SD | Mean | SD | Significant? | Summary | Adjusted P Value |
| Superior 4000 µm | 37,62778 | 3,626141 | 40,77333 | 3,641994 | No | ns | 0,3408 |
| Superior 500 µm | 55,90556 | 4,119337 | 56,81556 | 4,300474 | No | ns | > 0,9999 |
| Superior 250 µm | 58,03222 | 2,728194 | 57,97889 | 3,86317 | No | ns | > 0,9999 |
| Inferior 250 µm | 55,79556 | 2,94848 | 62,09444 | 4,231658 | Yes | ** | 0,0012 |
| Inferior 500 µm | 52,05555 | 2,86343 | 60,20111 | 2,840147 | Yes | **** | < 0,0001 |
| Inferior 4000 µm | 33,73111 | 3,24474 | 41,55667 | 2,46569 | Yes | **** | < 0,0001 |

**Comparism between different retinal positions**

**Lean**

| | Significant? | Summary | Adjusted P Value |
|---|---|---|---|
| Sup. 4000 µm vs. Sup. 500 µm | Yes | **** | < 0,0001 |
| Sup. 4000 µm vs. Sup. 250 µm | Yes | **** | < 0,0001 |
| Sup. 4000 µm vs. Inf. 250 µm | Yes | **** | < 0,0001 |
| Sup. 4000 µm vs. Inf. 500 µm | Yes | **** | < 0,0001 |
| Sup. 4000 µm vs. Inf. 4000 µm | No | ns | 0,2831 |
| Sup. 500 µm vs. Sup. 250 µm | No | ns | > 0,9999 |
| Sup. 500 µm vs. Inf. 250 µm | No | ns | > 0,9999 |
| Sup. 500 µm vs. Inf. 500 µm | No | ns | 0,3045 |
| Sup. 500 µm vs. Inf. 4000 µm | Yes | **** | < 0,0001 |
| Sup. 250 µm vs. Inf. 250 µm | No | ns | > 0,9999 |
| Sup. 250 µm vs. Inf. 500 µm | Yes | ** | 0,0061 |
| Sup. 250 µm vs. Inf. 4000 µm | Yes | **** | < 0,0001 |
| Inf. 250 µm vs. Inf. 500 µm | No | ns | 0,3609 |
| Inf. 250 µm vs. Inf. 4000 µm | Yes | **** | < 0,0001 |
| Inf. 500 µm vs. Inf. 4000 µm | Yes | **** | < 0,0001 |

**diabetic**

| | Significant? | Summary | Adjusted P Value |
|---|---|---|---|
| Sup. 4000 µm vs. Sup. 500 µm | Yes | **** | < 0,0001 |
| Sup. 4000 µm vs. Sup. 250 µm | Yes | **** | < 0,0001 |
| Sup. 4000 µm vs. Inf. 250 µm | Yes | **** | < 0,0001 |
| Sup. 4000 µm vs. Inf. 500 µm | Yes | **** | < 0,0001 |
| Sup. 4000 µm vs. Inf. 4000 µm | No | ns | > 0,9999 |
| Sup. 500 µm vs. Sup. 250 µm | No | ns | > 0,9999 |
| Sup. 500 µm vs. Inf. 250 µm | Yes | * | 0,025 |
| Sup. 500 µm vs. Inf. 500 µm | No | ns | 0,6096 |
| Sup. 500 µm vs. Inf. 4000 µm | Yes | **** | < 0,0001 |
| Sup. 250 µm vs. Inf. 250 µm | No | ns | 0,1993 |
| Sup. 250 µm vs. Inf. 500 µm | No | ns | > 0,9999 |
| Sup. 250 µm vs. Inf. 4000 µm | Yes | **** | < 0,0001 |
| Inf. 250 µm vs. Inf. 500 µm | No | ns | > 0,9999 |
| Inf. 250 µm vs. Inf. 4000 µm | Yes | **** | < 0,0001 |
| Inf. 500 µm vs. Inf. 4000 µm | Yes | **** | < 0,0001 |

**Table S4. Supplementary data to the statistical analysis of the number of nuclei in the ONL columns**

| | Lean | | Diabetic | | Lean - Diabetic | | |
|---|---|---|---|---|---|---|---|
| | Mean | SD | Mean | SD | Significant? | Summary | Adjusted P Value |
| Superior 4000 µm | 8,444445 | 0,5270463 | 8,333333 | 0,8660254 | No | ns | > 0,9999 |
| Superior 500 µm | 12 | 0,8660254 | 11,66667 | 1,322876 | No | ns | > 0,9999 |
| Superior 250 µm | 12,11111 | 0,3333333 | 11,88889 | 0,781736 | No | ns | > 0,9999 |
| Inferior 250 µm | 12 | 0,5 | 12,88889 | 0,781736 | No | ns | 0,0606 |
| Inferior 500 µm | 11,55556 | 0,5270463 | 12,22222 | 0,6666667 | No | ns | 0,3115 |
| Inferior 4000 µm | 8 | 0,5 | 8,888889 | 0,3333333 | No | ns | 0,0606 |

**Comparism between different retinal positions**

**Lean**

| | Significant? | Summary | Adjusted P Value |
|---|---|---|---|
| Sup. 4000 µm vs. Sup. 500 µm | Yes | **** | < 0,0001 |
| Sup. 4000 µm vs. Sup. 250 µm | Yes | **** | < 0,0001 |
| Sup. 4000 µm vs. Inf. 250 µm | Yes | **** | < 0,0001 |
| Sup. 4000 µm vs. Inf. 500 µm | Yes | **** | < 0,0001 |
| Sup. 4000 µm vs. Inf. 4000 µm | No | ns | > 0,9999 |
| Sup. 500 µm vs. Sup. 250 µm | No | ns | > 0,9999 |
| Sup. 500 µm vs. Inf. 250 µm | No | ns | > 0,9999 |
| Sup. 500 µm vs. Inf. 500 µm | No | ns | > 0,9999 |
| Sup. 500 µm vs. Inf. 4000 µm | Yes | **** | < 0,0001 |
| Sup. 250 µm vs. Inf. 250 µm | No | ns | > 0,9999 |
| Sup. 250 µm vs. Inf. 500 µm | No | ns | > 0,9999 |
| Sup. 250 µm vs. Inf. 4000 µm | Yes | **** | < 0,0001 |
| Inf. 250 µm vs. Inf. 500 µm | No | ns | > 0,9999 |
| Inf. 250 µm vs. Inf. 4000 µm | Yes | **** | < 0,0001 |
| Inf. 500 µm vs. Inf. 4000 µm | Yes | **** | < 0,0001 |

**diabetic**

| | Significant? | Summary | Adjusted P Value |
|---|---|---|---|
| Sup. 4000 µm vs. Sup. 500 µm | Yes | **** | < 0,0001 |
| Sup. 4000 µm vs. Sup. 250 µm | Yes | **** | < 0,0001 |
| Sup. 4000 µm vs. Inf. 250 µm | Yes | **** | < 0,0001 |
| Sup. 4000 µm vs. Inf. 500 µm | Yes | **** | < 0,0001 |
| Sup. 4000 µm vs. Inf. 4000 µm | No | ns | > 0,9999 |
| Sup. 500 µm vs. Sup. 250 µm | No | ns | > 0,9999 |
| Sup. 500 µm vs. Inf. 250 µm | Yes | ** | 0,0074 |
| Sup. 500 µm vs. Inf. 500 µm | No | ns | > 0,9999 |
| Sup. 500 µm vs. Inf. 4000 µm | Yes | **** | < 0,0001 |
| Sup. 250 µm vs. Inf. 250 µm | No | ns | 0,0594 |
| Sup. 250 µm vs. Inf. 500 µm | No | ns | > 0,9999 |
| Sup. 250 µm vs. Inf. 4000 µm | Yes | **** | < 0,0001 |
| Inf. 250 µm vs. Inf. 500 µm | No | ns | 0,7787 |
| Inf. 250 µm vs. Inf. 4000 µm | Yes | **** | < 0,0001 |
| Inf. 500 µm vs. Inf. 4000 µm | Yes | **** | < 0,0001 |

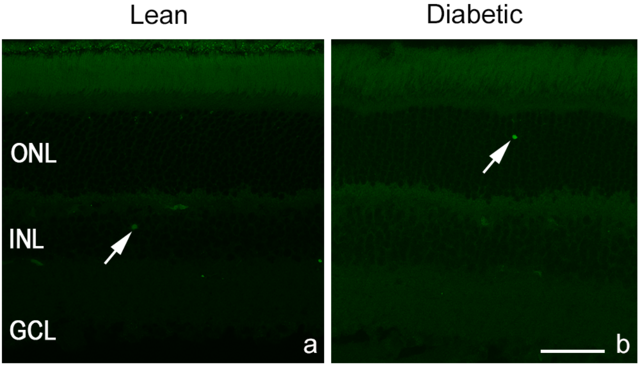


**Figure S5. Representative pictures of TUNEL labelled control and diabetic retinas.** Tunel-positive elements are marked by arrows. ONL: outer nuclear layer, INL: inner nuclear layer, GCL: ganglion cell layer. *Bar*: 20 μm.

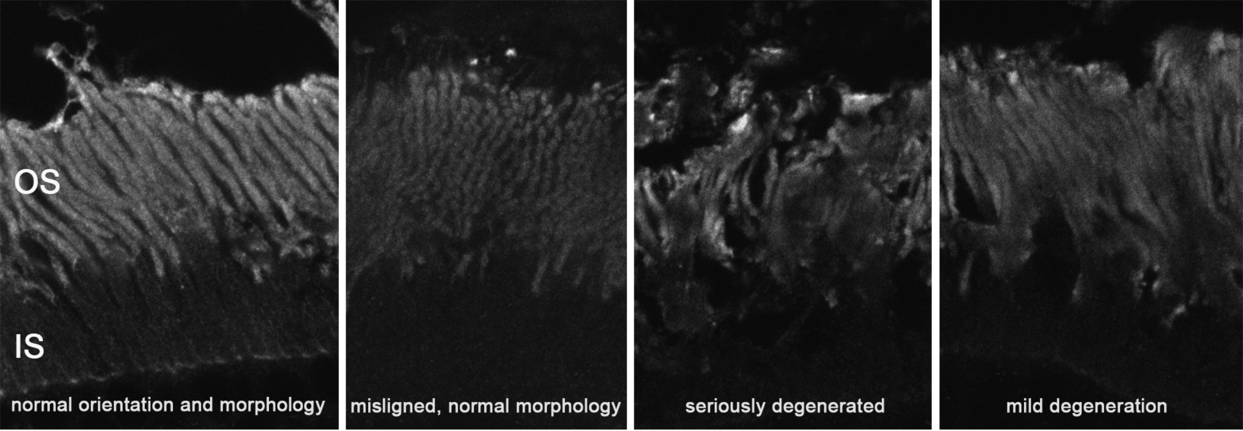


**Figure S6. Representative images from healthy and degenerated rod outer segments on single confocal planes.** OS: outer segments, IS: inner segments

**Table S7. Supplementary data to the statistical analysis of recoverin positive cone bipolar cells**

| | **Lean** | | **Diabetic** | | **Lean - Diabetic** | | |
|---|---|---|---|---|---|---|---|
| | Mean | SD | Mean | SD | Significant? | Summary | Adjusted P Value |
| Sup. periphery | 23,33333 | 6,914443 | 23,71428 | 4,858436 | No | ns | > 0,9999 |
| Sup. middle | 25,35714 | 6,789213 | 25,35714 | 2,373156 | No | ns | > 0,9999 |
| Sup. center | 25,8 | 5,942583 | 25,14286 | 5,111934 | No | ns | > 0,9999 |
| Inf. center | 25,92857 | 5,915615 | 25,14286 | 3,676477 | No | ns | > 0,9999 |
| Inf. middle | 26 | 6,380774 | 24,64286 | 3,953716 | No | ns | > 0,9999 |
| Inf. periphery | 22,53333 | 8,114068 | 22,07692 | 3,277742 | No | ns | > 0,9999 |

**Comparism between different retinal positions**

**Lean**

| | Significant? | Summary | Adjusted P Value |
|---|---|---|---|
| Sup. periphery vs. Sup. middle | No | ns | > 0,9999 |
| Sup. periphery vs. Sup. center | No | ns | > 0,9999 |
| Sup. periphery vs. Inf. center | No | ns | > 0,9999 |
| Sup. periphery vs. Inf. middle | No | ns | > 0,9999 |
| Sup. periphery vs. Inf. periphery | No | ns | > 0,9999 |
| Sup. middle vs. Sup. center | No | ns | > 0,9999 |
| Sup. middle vs. Inf. center | No | ns | > 0,9999 |
| Sup. middle vs. Inf. middle | No | ns | > 0,9999 |
| Sup. middle vs. Inf. periphery | No | ns | > 0,9999 |
| Sup. center vs. Inf. center | No | ns | > 0,9999 |
| Sup. center vs. Inf. middle | No | ns | > 0,9999 |
| Sup. center vs. Inf. periphery | No | ns | > 0,9999 |
| Inf. center vs. Inf. middle | No | ns | > 0,9999 |
| Inf. center vs. Inf. periphery | No | ns | > 0,9999 |
| Inf. middle vs. Inf. periphery | No | ns | > 0,9999 |

**diabetic**

| | Significant? | Summary | Adjusted P Value |
|---|---|---|---|
| Sup. periphery vs. Sup. middle | No | ns | > 0,9999 |
| Sup. periphery vs. Sup. center | No | ns | > 0,9999 |
| Sup. periphery vs. Inf. center | No | ns | > 0,9999 |
| Sup. periphery vs. Inf. middle | No | ns | > 0,9999 |
| Sup. periphery vs. Inf. periphery | No | ns | > 0,9999 |
| Sup. middle vs. Sup. center | No | ns | > 0,9999 |
| Sup. middle vs. Inf. center | No | ns | > 0,9999 |
| Sup. middle vs. Inf. middle | No | ns | > 0,9999 |
| Sup. middle vs. Inf. periphery | No | ns | > 0,9999 |
| Sup. center vs. Inf. center | No | ns | > 0,9999 |
| Sup. center vs. Inf. middle | No | ns | > 0,9999 |
| Sup. center vs. Inf. periphery | No | ns | > 0,9999 |
| Inf. center vs. Inf. middle | No | ns | > 0,9999 |
| Inf. center vs. Inf. periphery | No | ns | > 0,9999 |
| Inf. middle vs. Inf. periphery | No | ns | > 0,9999 |

**Table S8. Supplementary data to the statistical analysis of PKC-α positive amacrine cells in the INL**

| | Lean | | Diabetic | | Lean - Diabetic | | |
|---|---|---|---|---|---|---|---|
| | Mean | SD | Mean | SD | Significant? | Summary | Adjusted P Value |
| Sup. periphery | 1,090909 | 0,9438798 | 1,6 | 1,121224 | No | ns | > 0,9999 |
| Sup. middle | 3,545455 | 2,733629 | 3,785714 | 1,625687 | No | ns | > 0,9999 |
| Sup. center | 7,4 | 2,633122 | 4,7 | 1,337494 | Yes | ** | 0,0092 |
| Inf. center | 7,454545 | 1,507557 | 6,25 | 1,815339 | No | ns | 0,747 |
| Inf. middle | 6 | 1,843909 | 4,333333 | 1,58865 | No | ns | 0,1568 |
| Inf. periphery | 3,636364 | 3,042129 | 2 | 1,511858 | No | ns | 0,1736 |

**Comparism between different retinal positions**

**Lean**

| | Significant? | Summary | Adjusted P Value |
|---|---|---|---|
| Sup. periphery vs. Sup. middle | Yes | * | 0,0372 |
| Sup. periphery vs. Sup. center | Yes | **** | < 0,0001 |
| Sup. periphery vs. Inf. center | Yes | **** | < 0,0001 |
| Sup. periphery vs. Inf. middle | Yes | **** | < 0,0001 |
| Sup. periphery vs. Inf. periphery | Yes | * | 0,0259 |
| Sup. middle vs. Sup. center | Yes | **** | < 0,0001 |
| Sup. middle vs. Inf. center | Yes | **** | < 0,0001 |
| Sup. middle vs. Inf. middle | Yes | * | 0,0372 |
| Sup. middle vs. Inf. periphery | No | ns | > 0,9999 |
| Sup. center vs. Inf. center | No | ns | > 0,9999 |
| Sup. center vs. Inf. middle | No | ns | > 0,9999 |
| Sup. center vs. Inf. periphery | Yes | *** | 0,0001 |
| Inf. center vs. Inf. middle | No | ns | > 0,9999 |
| Inf. center vs. Inf. periphery | Yes | **** | < 0,0001 |
| Inf. middle vs. Inf. periphery | No | ns | 0,053 |

**diabetic**

| | Significant? | Summary | Adjusted P Value |
|---|---|---|---|
| Sup. periphery vs. Sup. middle | Yes | * | 0,0301 |
| Sup. periphery vs. Sup. center | Yes | ** | 0,0012 |
| Sup. periphery vs. Inf. center | Yes | **** | < 0,0001 |
| Sup. periphery vs. Inf. middle | Yes | ** | 0,0015 |
| Sup. periphery vs. Inf. periphery | No | ns | > 0,9999 |
| Sup. middle vs. Sup. center | No | ns | > 0,9999 |
| Sup. middle vs. Inf. center | Yes | * | 0,0155 |
| Sup. middle vs. Inf. middle | No | ns | > 0,9999 |
| Sup. middle vs. Inf. periphery | No | ns | 0,167 |
| Sup. center vs. Inf. center | No | ns | 0,8186 |
| Sup. center vs. Inf. middle | No | ns | > 0,9999 |
| Sup. center vs. Inf. periphery | Yes | ** | 0,0082 |
| Inf. center vs. Inf. middle | No | ns | 0,1349 |
| Inf. center vs. Inf. periphery | Yes | **** | < 0,0001 |
| Inf. middle vs. Inf. periphery | Yes | * | 0,0123 |

**Table S9. Supplementary data to the statistical analysis of PKC-α positive amacrine cells in the GCL**

| | Lean | | Diabetic | | Lean - Diabetic | | |
|---|---|---|---|---|---|---|---|
| | Mean | SD | Mean | SD | Significant? | Summary | Adjusted P Value |
| Sup. periphery | 3,545455 | 3,173756 | 4 | 2,374103 | No | ns | > 0,9999 |
| Sup. middle | 4 | 2,296242 | 9 | 3,247377 | Yes | *** | 0,0001 |
| Sup. center | 3,363636 | 2,01359 | 9,6 | 3,777124 | Yes | **** | < 0,0001 |
| Inf. center | 4,272727 | 2,327699 | 7,5 | 3,205897 | No | ns | 0,0549 |
| Inf. middle | 4,181818 | 2,227922 | 7,363636 | 2,766685 | No | ns | 0,0509 |
| Inf. periphery | 2,777778 | 2,333333 | 4,454545 | 3,20511 | No | ns | > 0,9999 |

**Comparism between different retinal positions**

**Lean**

| | Significant? | Summary | Adjusted P Value |
|---|---|---|---|
| Sup. periphery vs. Sup. middle | No | ns | > 0,9999 |
| Sup. periphery vs. Sup. center | No | ns | > 0,9999 |
| Sup. periphery vs. Inf. center | No | ns | > 0,9999 |
| Sup. periphery vs. Inf. middle | No | ns | > 0,9999 |
| Sup. periphery vs. Inf. periphery | No | ns | > 0,9999 |
| Sup. middle vs. Sup. center | No | ns | > 0,9999 |
| Sup. middle vs. Inf. center | No | ns | > 0,9999 |
| Sup. middle vs. Inf. middle | No | ns | > 0,9999 |
| Sup. middle vs. Inf. periphery | No | ns | > 0,9999 |
| Sup. center vs. Inf. center | No | ns | > 0,9999 |
| Sup. center vs. Inf. middle | No | ns | > 0,9999 |
| Sup. center vs. Inf. periphery | No | ns | > 0,9999 |
| Inf. center vs. Inf. middle | No | ns | > 0,9999 |
| Inf. center vs. Inf. periphery | No | ns | > 0,9999 |
| Inf. middle vs. Inf. periphery | No | ns | > 0,9999 |

**diabetic**

| | Significant? | Summary | Adjusted P Value |
|---|---|---|---|
| Sup. periphery vs. Sup. middle | Yes | *** | 0,0004 |
| Sup. periphery vs. Sup. center | Yes | *** | 0,0001 |
| Sup. periphery vs. Inf. center | No | ns | 0,0606 |
| Sup. periphery vs. Inf. middle | No | ns | 0,0686 |
| Sup. periphery vs. Inf. periphery | No | ns | > 0,9999 |
| Sup. middle vs. Sup. center | No | ns | > 0,9999 |
| Sup. middle vs. Inf. center | No | ns | > 0,9999 |
| Sup. middle vs. Inf. middle | No | ns | > 0,9999 |
| Sup. middle vs. Inf. periphery | Yes | ** | 0,0023 |
| Sup. center vs. Inf. center | No | ns | > 0,9999 |
| Sup. center vs. Inf. middle | No | ns | > 0,9999 |
| Sup. center vs. Inf. periphery | Yes | *** | 0,0007 |
| Inf. center vs. Inf. middle | No | ns | > 0,9999 |
| Inf. center vs. Inf. periphery | No | ns | 0,2066 |
| Inf. middle vs. Inf. periphery | No | ns | 0,2378 |

**Table S10. Supplementary data to the statistical analysis of parvalbumin positive AII amacrine cells**

| | Lean | | Diabetic | | Lean - Diabetic | | |
|---|---|---|---|---|---|---|---|
| | Mean | SD | Mean | SD | Significant? | Summary | Adjusted P Value |
| Sup. periphery | 19,375 | 1,59799 | 15,875 | 2,217356 | Yes | ** | 0,0033 |
| Sup. middle | 22 | 1,85164 | 22,4375 | 2,82769 | No | ns | > 0,9999 |
| Sup. center | 23,625 | 1,92261 | 22,46667 | 1,807392 | No | ns | > 0,9999 |
| Inf. center | 23,625 | 2,445842 | 23,46667 | 1,505545 | No | ns | > 0,9999 |
| Inf. middle | 24,125 | 2,799872 | 20,9375 | 2,322893 | Yes | ** | 0,0094 |
| Inf. periphery | 19,42857 | 2,992053 | 18 | 2,581989 | No | ns | > 0,9999 |

**Comparism between different retinal positions**

**Lean**

| | Significant? | Summary | Adjusted P Value |
|---|---|---|---|
| Sup. periphery vs. Sup. middle | No | ns | 0,3431 |
| Sup. periphery vs. Sup. center | Yes | ** | 0,0043 |
| Sup. periphery vs. Inf. center | Yes | ** | 0,0043 |
| Sup. periphery vs. Inf. middle | Yes | *** | 0,0008 |
| Sup. periphery vs. Inf. periphery | No | ns | > 0,9999 |
| Sup. middle vs. Sup. center | No | ns | > 0,9999 |
| Sup. middle vs. Inf. center | No | ns | > 0,9999 |
| Sup. middle vs. Inf. middle | No | ns | 0,968 |
| Sup. middle vs. Inf. periphery | No | ns | 0,4665 |
| Sup. center vs. Inf. center | No | ns | > 0,9999 |
| Sup. center vs. Inf. middle | No | ns | > 0,9999 |
| Sup. center vs. Inf. periphery | Yes | ** | 0,0079 |
| Inf. center vs. Inf. middle | No | ns | > 0,9999 |
| Inf. center vs. Inf. periphery | Yes | ** | 0,0079 |
| Inf. middle vs. Inf. periphery | Yes | ** | 0,0017 |

**diabetic**

| | Significant? | Summary | Adjusted P Value |
|---|---|---|---|
| Sup. periphery vs. Sup. middle | Yes | **** | < 0,0001 |
| Sup. periphery vs. Sup. center | Yes | **** | < 0,0001 |
| Sup. periphery vs. Inf. center | Yes | **** | < 0,0001 |
| Sup. periphery vs. Inf. middle | Yes | **** | < 0,0001 |
| Sup. periphery vs. Inf. periphery | No | ns | 0,141 |
| Sup. middle vs. Sup. center | No | ns | > 0,9999 |
| Sup. middle vs. Inf. center | No | ns | > 0,9999 |
| Sup. middle vs. Inf. middle | No | ns | 0,9749 |
| Sup. middle vs. Inf. periphery | Yes | **** | < 0,0001 |
| Sup. center vs. Inf. center | No | ns | > 0,9999 |
| Sup. center vs. Inf. middle | No | ns | 0,9635 |
| Sup. center vs. Inf. periphery | Yes | **** | < 0,0001 |
| Inf. center vs. Inf. middle | Yes | * | 0,0371 |
| Inf. center vs. Inf. periphery | Yes | **** | < 0,0001 |
| Inf. middle vs. Inf. periphery | Yes | ** | 0,0058 |

**Table S11. Supplementary data to the statistical analysis of calretinin positive cells in the INL**

| | Lean | | Diabetic | | Lean - Diabetic | | |
|---|---|---|---|---|---|---|---|
| | Mean | SD | Mean | SD | Significant? | Summary | Adjusted P Value |
| Sup. periphery | 36,6875 | 6,935116 | 39,1875 | 7,41367 | No | ns | > 0,9999 |
| Sup. middle | 41,5625 | 5,427937 | 42,3125 | 8,187134 | No | ns | > 0,9999 |
| Sup. center | 44,5 | 7,247988 | 44,5 | 8,445907 | No | ns | > 0,9999 |
| Inf. center | 40,25 | 5,927338 | 43,6875 | 6,161372 | No | ns | > 0,9999 |
| Inf. middle | 42,3125 | 7,734931 | 40,75 | 6,952218 | No | ns | > 0,9999 |
| Inf. periphery | 38,93333 | 8,680575 | 42,0625 | 6,627908 | No | ns | > 0,9999 |

**Comparism between different retinal positions**

**Lean**

| | Significant? | Summary | Adjusted P Value |
|---|---|---|---|
| Sup. periphery vs. Sup. middle | No | ns | 0,8571 |
| Sup. periphery vs. Sup. center | Yes | * | 0,0373 |
| Sup. periphery vs. Inf. center | No | ns | > 0,9999 |
| Sup. periphery vs. Inf. middle | No | ns | 0,4265 |
| Sup. periphery vs. Inf. periphery | No | ns | > 0,9999 |
| Sup. middle vs. Sup. center | No | ns | > 0,9999 |
| Sup. middle vs. Inf. center | No | ns | > 0,9999 |
| Sup. middle vs. Inf. middle | No | ns | > 0,9999 |
| Sup. middle vs. Inf. periphery | No | ns | > 0,9999 |
| Sup. center vs. Inf. center | No | ns | > 0,9999 |
| Sup. center vs. Inf. middle | No | ns | > 0,9999 |
| Sup. center vs. Inf. periphery | No | ns | 0,4926 |
| Inf. center vs. Inf. middle | No | ns | > 0,9999 |
| Inf. center vs. Inf. periphery | No | ns | > 0,9999 |
| Inf. middle vs. Inf. periphery | No | ns | > 0,9999 |

**diabetic**

| | Significant? | Summary | Adjusted P Value |
|---|---|---|---|
| Sup. periphery vs. Sup. middle | No | ns | > 0,9999 |
| Sup. periphery vs. Sup. center | No | ns | 0,5754 |
| Sup. periphery vs. Inf. center | No | ns | > 0,9999 |
| Sup. periphery vs. Inf. middle | No | ns | > 0,9999 |
| Sup. periphery vs. Inf. periphery | No | ns | > 0,9999 |
| Sup. middle vs. Sup. center | No | ns | > 0,9999 |
| Sup. middle vs. Inf. center | No | ns | > 0,9999 |
| Sup. middle vs. Inf. middle | No | ns | > 0,9999 |
| Sup. middle vs. Inf. periphery | No | ns | > 0,9999 |
| Sup. center vs. Inf. center | No | ns | > 0,9999 |
| Sup. center vs. Inf. middle | No | ns | > 0,9999 |
| Sup. center vs. Inf. periphery | No | ns | > 0,9999 |
| Inf. center vs. Inf. middle | No | ns | > 0,9999 |
| Inf. center vs. Inf. periphery | No | ns | > 0,9999 |
| Inf. middle vs. Inf. periphery | No | ns | > 0,9999 |

**Table S12. Supplementary data to the statistical analysis of calretinin positive cells in the GCL**

| | Lean | | Diabetic | | Lean - Diabetic | | |
|---|---|---|---|---|---|---|---|
| | Mean | SD | Mean | SD | Significant? | Summary | Adjusted P Value |
| Sup. periphery | 15,875 | 4,395073 | 15,5 | 6,044281 | No | ns | > 0,9999 |
| Sup. middle | 17,625 | 5,560276 | 20,125 | 6,108737 | No | ns | > 0,9999 |
| Sup. center | 20,25 | 6,668333 | 21,3125 | 4,686417 | No | ns | > 0,9999 |
| Inf. center | 19,4375 | 4,049177 | 24,0625 | 4,296801 | No | ns | 0,0695 |
| Inf. middle | 20,5 | 4,516636 | 20,8125 | 4,415409 | No | ns | > 0,9999 |
| Inf. periphery | 17,46667 | 5,31664 | 18,0625 | 4,711246 | No | ns | > 0,9999 |

**Comparism between different retinal positions**

**Lean**

| | Significant? | Summary | Adjusted P Value |
|---|---|---|---|
| Sup. periphery vs. Sup. middle | No | ns | > 0,9999 |
| Sup. periphery vs. Sup. center | No | ns | 0,2525 |
| Sup. periphery vs. Inf. center | No | ns | 0,7647 |
| Sup. periphery vs. Inf. middle | No | ns | 0,1738 |
| Sup. periphery vs. Inf. periphery | No | ns | > 0,9999 |
| Sup. middle vs. Sup. center | No | ns | > 0,9999 |
| Sup. middle vs. Inf. center | No | ns | > 0,9999 |
| Sup. middle vs. Inf. middle | No | ns | > 0,9999 |
| Sup. middle vs. Inf. periphery | No | ns | > 0,9999 |
| Sup. center vs. Inf. center | No | ns | > 0,9999 |
| Sup. center vs. Inf. middle | No | ns | > 0,9999 |
| Sup. center vs. Inf. periphery | No | ns | > 0,9999 |
| Inf. center vs. Inf. middle | No | ns | > 0,9999 |
| Inf. center vs. Inf. periphery | No | ns | > 0,9999 |
| Inf. middle vs. Inf. periphery | No | ns | > 0,9999 |

**diabetic**

| | Significant? | Summary | Adjusted P Value |
|---|---|---|---|
| Sup. periphery vs. Sup. middle | No | ns | 0,1738 |
| Sup. periphery vs. Sup. center | Yes | * | 0,0239 |
| Sup. periphery vs. Inf. center | Yes | **** | < 0,0001 |
| Sup. periphery vs. Inf. middle | No | ns | 0,0575 |
| Sup. periphery vs. Inf. periphery | No | ns | > 0,9999 |
| Sup. middle vs. Sup. center | No | ns | > 0,9999 |
| Sup. middle vs. Inf. center | No | ns | 0,468 |
| Sup. middle vs. Inf. middle | No | ns | > 0,9999 |
| Sup. middle vs. Inf. periphery | No | ns | > 0,9999 |
| Sup. center vs. Inf. center | No | ns | > 0,9999 |
| Sup. center vs. Inf. middle | No | ns | > 0,9999 |
| Sup. center vs. Inf. periphery | No | ns | > 0,9999 |
| Inf. center vs. Inf. middle | No | ns | > 0,9999 |
| Inf. center vs. Inf. periphery | Yes | * | 0,017 |
| Inf. middle vs. Inf. periphery | No | ns | > 0,9999 |
